# Fibroblast growth factor 2 acts as an upstream regulator of inhibition of pulmonary fibroblast activation

**DOI:** 10.1002/2211-5463.13691

**Published:** 2023-08-17

**Authors:** Xiangqin Tian, Yangyang Jia, Yonglong Guo, Hongyin Liu, Xinhua Cai, Yong Li, Zhuangzhuang Tian, Changye Sun

**Affiliations:** ^1^ Henan Key Laboratory of Medical Tissue Regeneration Xinxiang Medical University China; ^2^ Department of Cardiology, The First Affiliated Hospital Xinxiang Medical University China; ^3^ Department of Biochemistry, Institute of Systems, Molecular and Integrative Biology University of Liverpool UK

**Keywords:** extracellular matrix, FGF2, mRNA sequencing, pulmonary fibroblasts, α‐SMA

## Abstract

Fibroblast growth factor (FGF) signaling plays a crucial role in lung development and repair. Fibroblast growth factor 2 (FGF2) can inhibit fibrotic gene expression and suppress the differentiation of pulmonary fibroblasts (PFs) into myofibroblasts *in vitro*, suggesting that FGF2 is a potential target for inhibiting pulmonary fibrosis. To gain deeper insights into the molecular mechanism underlying FGF2‐mediated regulation of PFs, we performed mRNA sequencing analysis to systematically and globally uncover the regulated genes and biological functions of FGF2 in PFs. Gene Ontology analysis revealed that the differentially expressed genes regulated by FGF2 were enriched in multiple cellular functions including extracellular matrix (ECM) organization, cytoskeleton formation, β‐catenin‐independent Wnt signaling pathway, supramolecular fiber organization, epithelial cell proliferation, and cell adhesion. Gene Set Enrichment Analysis and cellular experiments confirmed that FGF2 can suppress ECM and actin filament organization and increase PFs proliferation. Taken together, these findings indicate that FGF2 acts as an upstream regulator of the inhibition of PFs activation and may play a regulatory role in pulmonary fibrosis.

## Graphical abstract

Fibroblast growth factor 2 (FGF2) can suppress pulmonary fibroblast (PF) activation *in vitro*. In this study, mRNA sequencing was used to analyze the differentially expressed genes regulated by FGF2: 172 upregulated genes and 490 downregulated genes. Gene enrichment analysis revealed that FGF2 can regulate multiple biological functions of PFs, including extracellular matrix organization, actin filament organization, and cell proliferation.

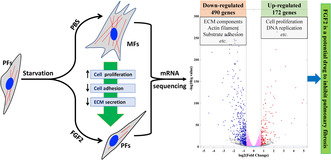

**Abbreviations**
BP: biological process
CFSE: 5(6)‐Carboxyfluorescein diacetate *N*‐succinimidyl ester
DDR2: discoidin domain receptor 2
DEGs: differentially expressed genes
DMEM: Dulbecco's modified Eagle's medium
ECM: extracellular matrix
FGF: fibroblast growth factor
GO: gene ontology
GSEA: Gene Set Enrichment Analysis
LMW: low molecular weight FGF2
MAPK: mitogen‐activated protein kinase kinase
MMPs: matrix metalloproteinases
PFs: pulmonary fibroblasts
TBS: tris‐buffered saline
TGFβ1: transforming growth factor‐β 1
α‐SMA: alpha smooth muscle actin

The lung is an organ contacting directly with the external environment, which is threatened by many substances in the air, for example, airborne pathogens, pollutants, particles, cigarette smoke, and other toxic byproducts arising from fuel combustion and various industries [1]. Most lung diseases, including pulmonary hypertension, obstructive sleep apnea, rheumatoid arthritis, systemic sclerosis, and coronavirus disease (COVID‐19) [2], can lead to lung fibrosis [3]. Lung fibrosis is characterized by fibroblast migration, proliferation, and differentiation into myofibroblast, also known as fibroblast activation. During this process, the myofibroblasts produce high levels of alpha smooth muscle actin (α‐SMA) and extracellular matrix (ECM) proteins, including type I and III collagen [4]. Therefore, inhibiting the activation of pulmonary fibroblasts (PFs) is crucial in preventing lung fibrosis.

Fibroblast growth factors (FGFs) are associated closely with development and pathogenesis of the lung [5]. FGF2 and its receptor (FGFR) are highly expressed in the lung fibrosis tissues [6]. Two different isoforms of FGF2 were identified, low molecular weight FGF2 (LMW FGF2: 18 kDa) and high molecular weight FGF2 (HMW FGF2: 22, 22.5, and 24 kDa) [7]. LMW FGF2 is generally used as a recombinant protein drug in the therapeutics of tissue repair. Previous studies have found that FGF2 has the ability to stimulate the migration and proliferation of PFs [8, 9]. Guzy and Xiao found that FGF2 is required for epithelial recovery following bleomycin‐induced lung injury in mice, but not for pulmonary fibrosis [10, 11]. Meanwhile, Koo *et al*. [12] found that FGF2 has antifibrotic effect on the inhibition of collagen production and PFs activation.

FGF2 can activate multiple signaling pathways, for example, mitogen‐activated protein kinase kinase (MAPK), ERK1/2, AKT, STAT3, and phospholipase Cγ, to regulate cell differentiation, proliferation, and migration [13, 14, 15]. In the previous studies, it was found that FGF2 could suppress PFs activation induced by transforming growth factor‐β 1 (TGFβ1) and inhibit profibrotic protein expression through activating the MEK/ERK pathway [12, 16]. The suppressive effect of FGF2 could be blocked by inhibition of FGFR1 with its siRNA or ERK1/2 with U0126 inhibitor [12, 16]. These findings emphasize the potential importance of FGF2 in the therapeutic of lung fibrosis, but further investigation is required to elucidate its precise role in the process.

In this study, we conducted a comprehensive analysis, involving transcriptomics, morphology, and functional assessment, to investigate the impact of recombinant FGF2 protein drug on PFs activation and the other relevant biological functions, which would provide a basis for the clinical transition of FGF2 in the treatment of lung repair.

## Methods

### Reagents

In this study, anti‐alpha smooth muscle actin (ab7817), anti‐discoidin domain receptor 2 (DDR2) antibody (ab221812), anti‐collagen type I antibody (ab34710), and anti‐collagen type III antibody (ab7778) were purchased from Abcam company (Shanghai, China), and anti‐ERK1/2 (#4695) and anti‐p‐ERK1/2 (#5726) were purchased from Cell Signaling Technology (Danvers, MA, USA). Recombinant TGF‐β1 and U0126 were ordered from Sigma‐Aldrich (St. Louis, MO, USA). Recombinant FGF2 (18 kDa) with a hexa‐histidine tag was prepared as described [17, 18]. The purified FGF2 was diluted to 20 μg·mL^−1^ with PBS for further experiments.

### Animals

Neonatal Sprague–Dawley rats (1–3 days old) were obtained from Beijing Vital River Laboratory Animal Technology Co., Ltd (Beijing, China), and animal experiments were approved by the XXMU Animal Supervision Committee (approval no. XYLL‐2021‐033). All animals were maintained under pathogen‐free conditions and stored humanely at 23 °C under a 12‐h light/12‐h dark cycle with free access to food and water.

### Pulmonary fibroblasts isolation and culture

Lungs were isolated from neonatal Sprague–Dawley rats to prepare PFs using the differential adherent properties of fibroblasts as previously described with minor modifications [19]. Briefly, neonatal rats were euthanized by CO_2_ asphyxiation and the lungs were excised under sterile conditions. The isolated lungs were cut into approximately 1 mm^3^ piece and washed with Hanks' balanced salt solution. The prepared tissues were digested with collagenase type II and trypsin for 20 min at 37 °C in a shaking bath. The degradation was stopped by the addition of Dulbecco's modified Eagle's medium (DMEM; Hyclone, Guangzhou, China) supplemented with 10% (v/v) FBS (Gibco, Auckland, New Zealand) and 1% penicillin/streptomycin. The supernatant was filtered with a 70 μm cell strainer and centrifuged at 1000 ***g*** for 5 min to pellet the isolated cells. The cell pellet was resuspended with DMEM containing 10% FBS and 1% penicillin/streptomycin and cultured in a tissue culture plate for 45 min. The floating cells were removed and the PFs attached to the plate were washed twice with PBS. The isolated PFs were cultured at 37 °C with 5% CO_2_ for 24 h before further detection. The expression of DDR2 was determined to quantify the purity of PFs. The purified PFs were treated with various concentrations of FBS or FGF2 to study their regulatory effects on PFs, and TGF‐β1 (2 ng·mL^−1^) was applied to induce PFs activation to determine the inhibitory effect of FGF2 on TGF‐β1‐induced PFs activation.

### Immunofluorescence staining

Pulmonary fibroblasts were seeded in 24‐well plates at a density of 2 × 10^5^ cells·mL^−1^ for immunofluorescence staining. Briefly, PFs were fixed with 4% paraformaldehyde for 20 min when 80% confluence. After washing the cells three times with PBS, they were permeabilized with 0.1% Triton X‐100 for 20 min and incubated with appropriate primary antibodies (the antibodies against DDR2, α‐SMA, collagen type III, and collagen type I) at 4 °C overnight, and the second antibodies [FITC‐labeled goat anti‐rabbit or mouse IgG (H + L), 1 : 500] for 2 h at room temperature. Nuclei were stained with propidium iodide (Beyotime, Shanghai, China) for 30 min. Fluorescence images were captured with a laser scanning confocal microscope (FV1000; Olympus, Tokyo, Japan).

### Western blot analysis

Total protein from PFs was extracted by SDS lysis buffer. The protein samples were separated by 12.5% SDS/PAGE and transferred onto a polyvinylidene fluoride membrane (Millipore, Merck, Cork, Ireland). The membrane was blocked by tris‐buffered saline (TBS) containing 5% skimmed milk and incubated with primary antibodies (against α‐SMA, collagen type I, collagen type III, ERK1/2, p‐ERK1/2, and GAPDH) at 4 °C for 18 h. Then, the membranes were incubated with HRP‐conjugated secondary antibodies [collagen type I and III with HRP‐labeled goat anti‐rabbit IgG (H + L); α‐SMA and GAPDH with HRP‐labeled goat anti‐mouse IgG (H + L); Beyotime] at room temperature for 2 h. After washing the membranes three times with TBST (150 mm NaCl, 20 mm Tris‐base, 0.05% Tween‐20), protein bands were visualized on an ECL system (Amersham Biosciences, Piscataway, NJ, USA), and the results were analyzed using the image‐pro plus 6.0 software (Rockville, MA, USA).

### RNA extraction and quantitative real‐time PCR (qRT‐PCR)

The primers for the target genes were designed with oligo 6.0 software (Molecular Biology Insights, Inc., Cascade, CO, USA) and synthesized by GENEWIZ Company (Suzhou, China). The primer sequences of the target genes are provided in Table S1. The total RNAs were extracted with TRIzol reagent (Thermo Fisher Scientific, Shanghai, China). Reverse transcription and PCR amplification were conducted according to the RevertAid First Strand cDNA Synthesis kit (Thermo Fisher Scientific) and SYBR Premix Ex Taq™ II (TAKARA Bio Inc., Dalian, China) protocols, respectively. All the reactions during the process were amplified in triplicate on a QuantStudio™ 7 Flex Real‐Time PCR instrument (Applied Biosystems, Life Technologies, Woodlands, Singapore). β‐Actin was applied as the endogenous standard for subsequent analysis.

### RNA sequencing and analysis

RNA samples were collected for the FGF2 treatment group and the control group. Total RNA was extracted with an RNAiso Plus kit (TaKaRa, Osaka, Japan). Library construction and sequencing were performed by the GENEWIZ company. For gene expression analysis, the matched reads were normalized according to the exon model, and the differences in gene expression were analyzed with the deseq2 bioconductor package by the GENEWIZ company. The differently expressed genes (absolute value of log_2_(Fold Change) is over than 0.75, log_10_(baseMean) ≥ 2, and *q*‐value < 0.05) were screened and displayed in matlab (2019a), as described previously [20]. The relevant analyses, including Gene Ontology (GO) analysis, Gene Set Enrichment Analysis (GSEA), and KEGG analysis, were performed with clusterprofiler package in r and metascape (3.6) [21, 22].

### Proliferation assay

The proliferation of PFs was assessed by YF®488 Click‐iT EdU Imaging Kits (Useverbright, Suzhou, China) according to the manufacturer's instructions. Briefly, the cells were seeded in a 96‐well plate and incubated for 12 h in DMEM supplemented with 10% FBS, and then serum‐starved for 24 h. The cells were incubated with EdU solution for 16 h. After fixed with 4% paraformaldehyde for 30 min, the cells were permeabilized with 0.5% Triton X‐100 for 10 min. Then YF® 488 Azide was added to label the synthesized DNA and nuclei were stained with PI. All images were taken by a confocal microscope (FV1000). The proliferation ratio was analyzed by imagej (version 1.8.0) software (Bethesda, MA, USA).

### Cell adhesion assay

The PFs were placed in 24‐well plates at a density of 1.0 × 10^5^ cells/well and incubated with FGF2 for 24 h. The PFs in the both control group and FGF2 group were trypsinized for 30 s and fixed with 4% paraformaldehyde for 15 min. After washing three times with PBS, the cells were incubated with 5(6)‐Carboxyfluorescein diacetate *N*‐succinimidyl ester (CFSE; Sigma, Steinheim, Germany) to stain cellular proteins for 1 h at room temperature. Nuclei were stained with PI. All images were taken by a confocal microscope (FV1000). The cell‐spreading area was analyzed by imagej software.

### Statistical analysis

The data are presented as means ± SEM. graphpad prism software version 9.0 (GraphPad Software Inc., San Diego, CA, USA) was employed for statistical analyses. An unpaired Student's *t*‐test or one‐way ANOVA followed by Bonferroni's multiple‐comparisons *post hoc* test was used for statistical comparisons. Significance was determined based on the following criteria: *P < 0.05, **P < 0.01, and ***P < 0.001.

## Results

### Isolation of PFs and their activation *in vitro*

**Fig. 1:**
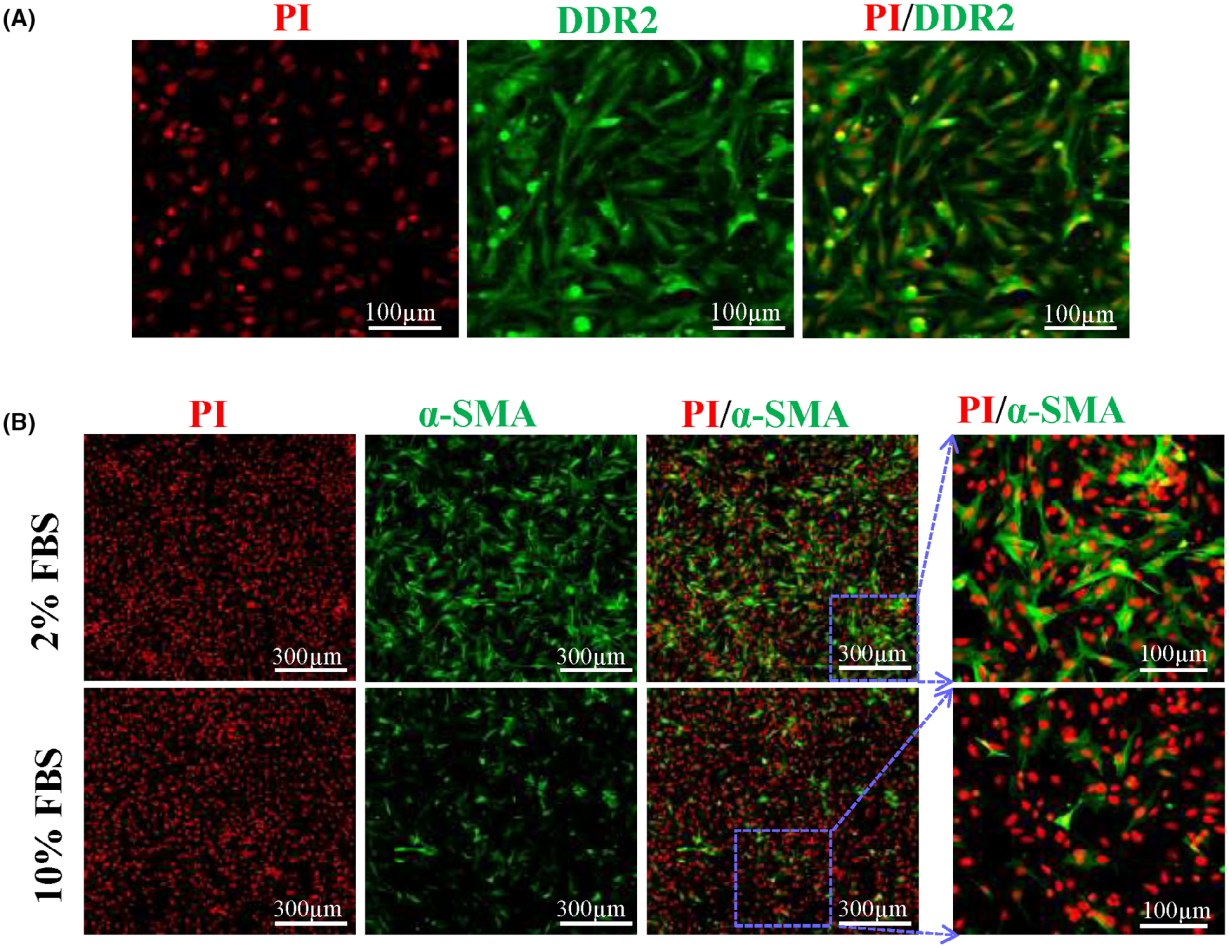
Identification of the rat PFs phenotype. (A) DDR2 immunostaining (green). Scale bar: 100 μm. (B) α‐SMA immunostaining (green) of PFs cultured in different concentrations of FBS. Scale bar: 100 and 300 μm.

The purity of PFs was identified by DDR2 staining, and the majority of the isolated cells were positively stained with DDR2 (Fig. 1A). PFs appeared typically spindle‐ or triangle‐like shapes under a light microscope (Fig. 1A). We investigated the expression of α‐SMA protein in PFs by immunofluorescence staining. As shown (Fig. 1B), α‐SMA was more expressed in PFs in 2.0% serum than that in 10% serum medium after 48 h of incubation, suggesting low serum could induce PFs activation. Therefore, we selected a 2.0% serum medium for subsequent experiments.

### Effect of FGF2 on PFs activation

**Fig. 2:**
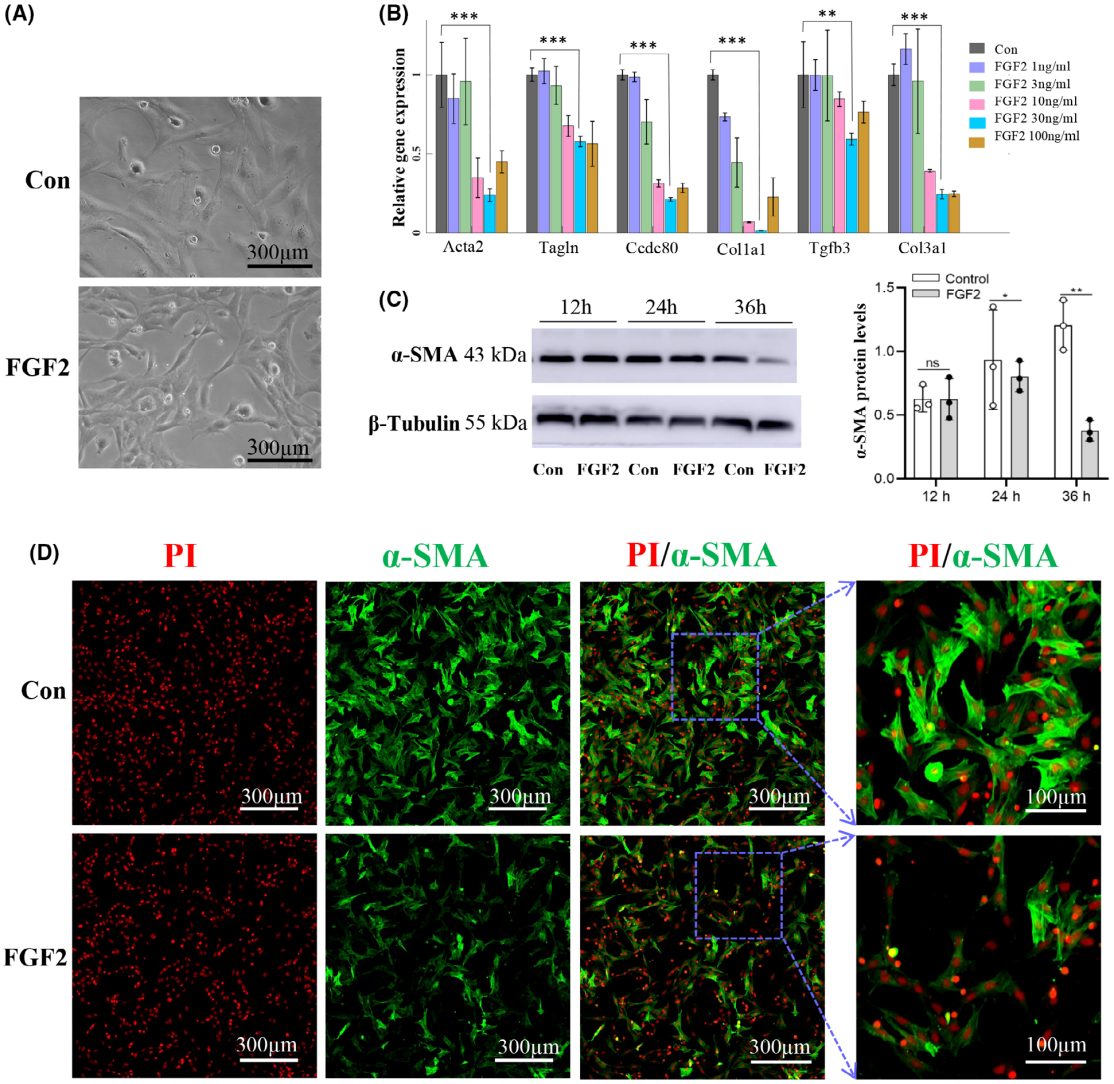
Representative characters of PFs differentiation after FGF2 treatment. (A) Different cell morphologies of PFs treated with and without FGF2. Scale bar: 300 μm. (B) The mRNAs expression of Acta2, Col1a1, Col3a1 Tgfb3, Tagln, and Ccdc80 in PFs cultured in different concentrations of FGF2. (C) The protein levels of α‐SMA were determined by western blot analysis. (D) α‐SMA protein showed more strongly expressed in the control group than that in the FGF2‐treated group by immunostaining. Scale bar: 100 and 300 μm. The data are presented as means ± SEM and analyzed using an unpaired Student's *t*‐test for statistical comparison (ns, no significant difference; *P < 0.05, **P < 0.01, and ***P < 0.001, *n* = 3).

The result showed that primary rat PFs *in vitro* display considerable change in cell morphology after FGF2 treatment (Fig. 2A). The cells exhibited an elongated, spindle‐shaped appearance in the FGF2‐treated group, while the cells in the control group are more flattened and stretched (Fig. 2A). We tested some genes expressions of *Acta2* (*α‐SMA*), *Col1a1*, *Col3a1 Tgfb3*, *Tagln*, and *Ccdc80* in PFs treated with different doses of FGF2 for 24 h. The results show that the transcription of fibroblast activation genes was dramatically decreased by 30 ng·mL^−1^ FGF2 (Fig. 2B). The expression level of α‐SMA was also significantly downregulated by 30 ng·mL^−1^ FGF2 (Fig. 2C,D). Based on these results, we applied 30 ng·mL^−1^ FGF2 to treat PFs for the subsequent experiments.

### The highly expressed genes and enriched functions analyzed by mRNA sequencing

**Fig. 3:**
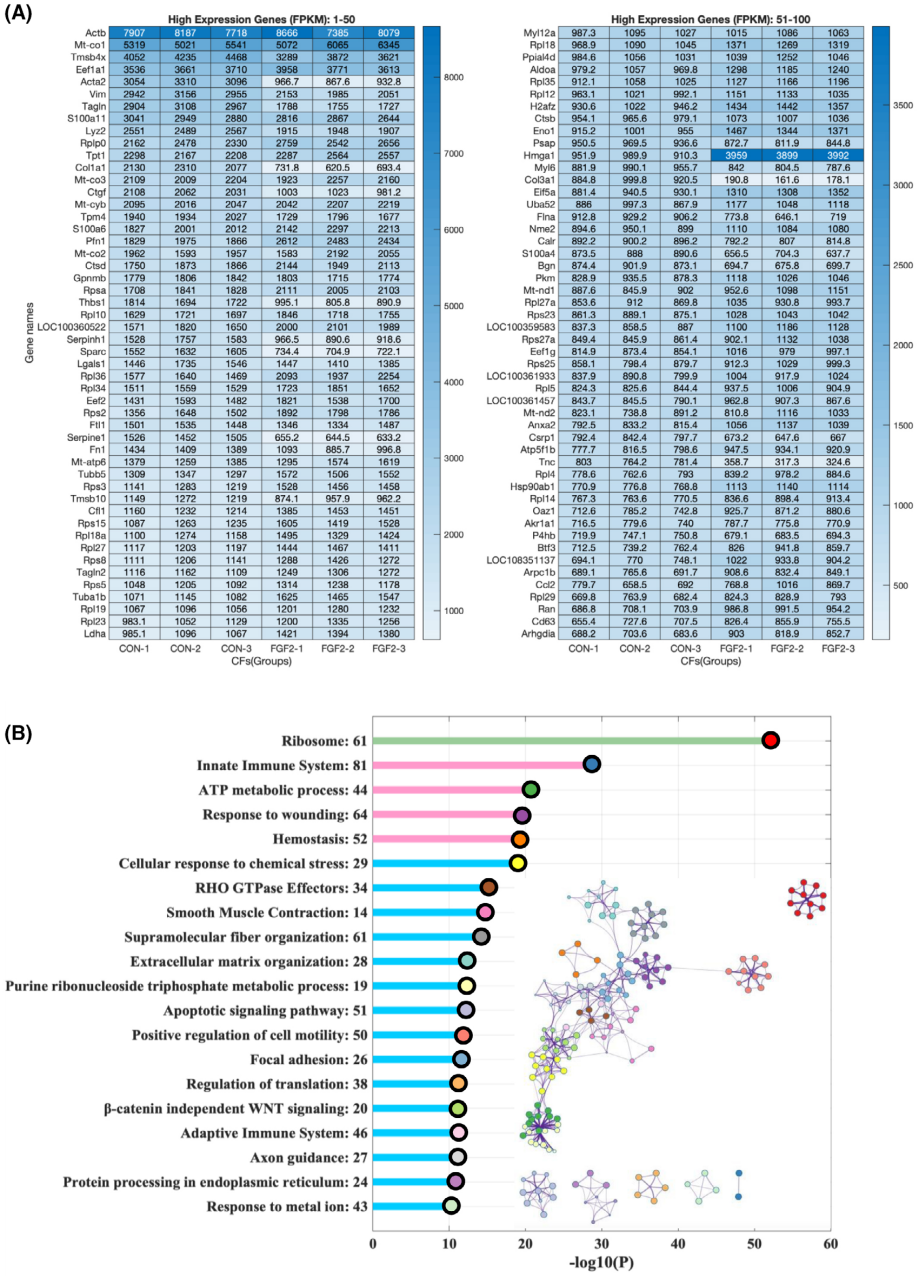
Highly expressed genes in PFs. (A) The mRNA expression levels in the PFs control group were sorted and the top 100 highly expressed genes are listed. (B) Enrichment analysis of the top 500 highly expressed genes shown in color code, and the metascape enrichment network visualization shows the cluster of enriched terms.

After treatment for 24 h, PFs treated with and without FGF2 were collected and the corresponding RNA samples were prepared for mRNA sequencing. The mRNA expression level was sorted, and the top 100 highly expressed genes are displayed (Fig. 3A). Most of these genes play important roles in PFs. There were many genes related to ECM proteins that are downregulated dramatically by FGF2, for example, *Acta2*, *Col1a1*, *Ctgf*, *Col3a1*, *Thbs1*, *Serprinh1*, *Sparc*, *Fn1*, and *Tnc* (Fig. 3A). We performed gene enrichment analyzed for the top 500 genes by metascape. The results demonstrate that the high expressed genes are enriched in the ribosome, innate immune system, ATP metabolic process, wounding, homeostasis, cellular response to chemical stress, smooth muscle contraction, ECM organization, focal adhesion, etc. (Fig. 3B), which reflect the biological activities of fibroblasts. In these enrichment networks, ECM organization, smooth muscle contraction, β‐catenin independent Wnt signaling, wounding, supramolecular fiber organization, and focal adhesion were closely related to tissue fibrosis (Fig. 3B). The differentially expressed genes (DEGs) involved in MAPK, PI3K‐AKT signaling, WNT signaling, and focal adhesion signaling are labeled and displayed in KEGG pathways (Figs S1–S4).

### Transcriptional profile of PFs treated with FGF2

**Fig. 4:**
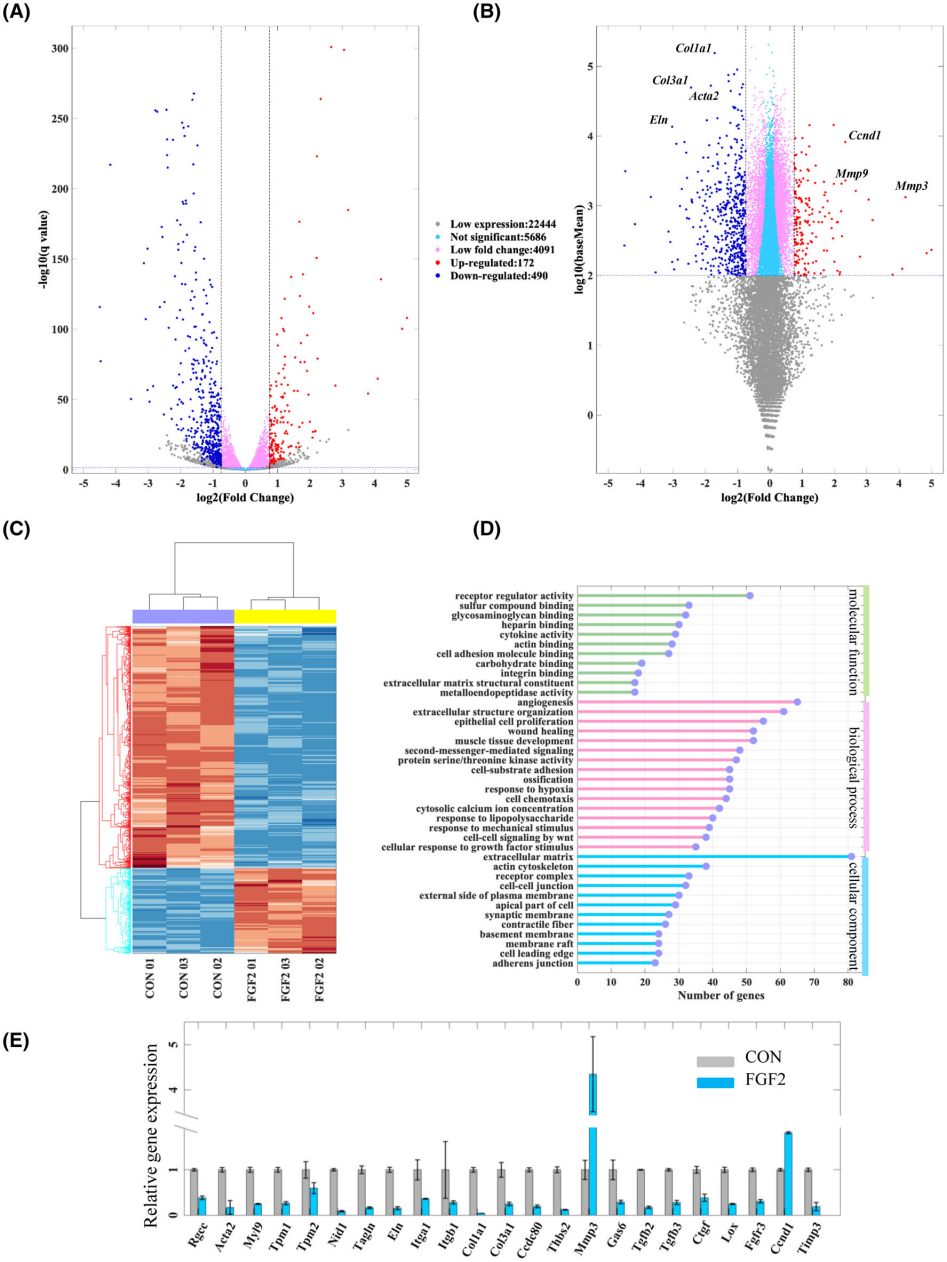
Transcriptional profiles of DEGs of PFs treated with‐ and without FGF2. (A, B) Volcano plot and fold change against expression plot represent differential gene expression. (C) Hierarchical clustering of DEGs of PFs of FGF2 group vs control group. (D) The top enriched functions of DEGs. (E) The relative expression levels of *Rgcc*, *Acta2*, *my19*, *Tmp2*, *Nidl*, *Eln*, *Itgα1*, *Itgb1*, *Col3a1*, *Cola1*, *Ccdc80*, *Thbs2*, *Mmp3*, *Gas6*, *Tgfb2*, *Tgfb3*, *Ctgf*, *Lox*, *Fgfr3*, *Ccnd1*, and *Timp3* were analyzed by qRT‐PCR. The data are presented as means ± SEM and analyzed using an unpaired Student's *t*‐test for statistical comparison (ns, no significant difference; *P < 0.05, **P < 0.01, *n* = 3).

The DEGs were screened out by limiting *P*‐value, and fold change. The results show that 172 genes are upregulated and 490 are downregulated (Fig. 4A,B), and the detail of these DEGs is shown in Table S2. It is shown that the expression profibrotic genes, for example, *Col1a1*, *Col3a1*, *Eln*, and *Acta2*, were decreased, while the expression of *Mmp* genes and *Ccnd1* was strongly increased (Fig. 4B). The expression levels of each sample were subjected to hierarchical clustering, and it was observed that the repeated samples exhibited high consistency (Fig. 4C). We performed GO classification and functional enrichment analysis to study the regulated functions by FGF2. The DEGs were identified to be significantly enriched in several functional terms, including ECM, collagen metabolic, and cell adhesion in groups of cellular component and biological process (BP; Fig. 4D).

### Validation of DEGs by qRT‐PCR

To verify the reliability and reproducibility of RNA sequencing results, we performed qRT‐PCR to determine partial DEGs, such as *Tgfb1*, *Tgfb2*, *Tgfb3*, and *Itgα*, *Itgβ*, *Eln*, *Col1*, *Col3*, *Ccdc80*, and *Mmp3*. The results of the qRT‐PCR analysis showed the same expression trends as that of RNA sequencing, indicating the reliability of transcriptomic data used for the analysis in the present study (Fig. 4E).

### Enrichment analysis for DEGs

**Fig. 5:**
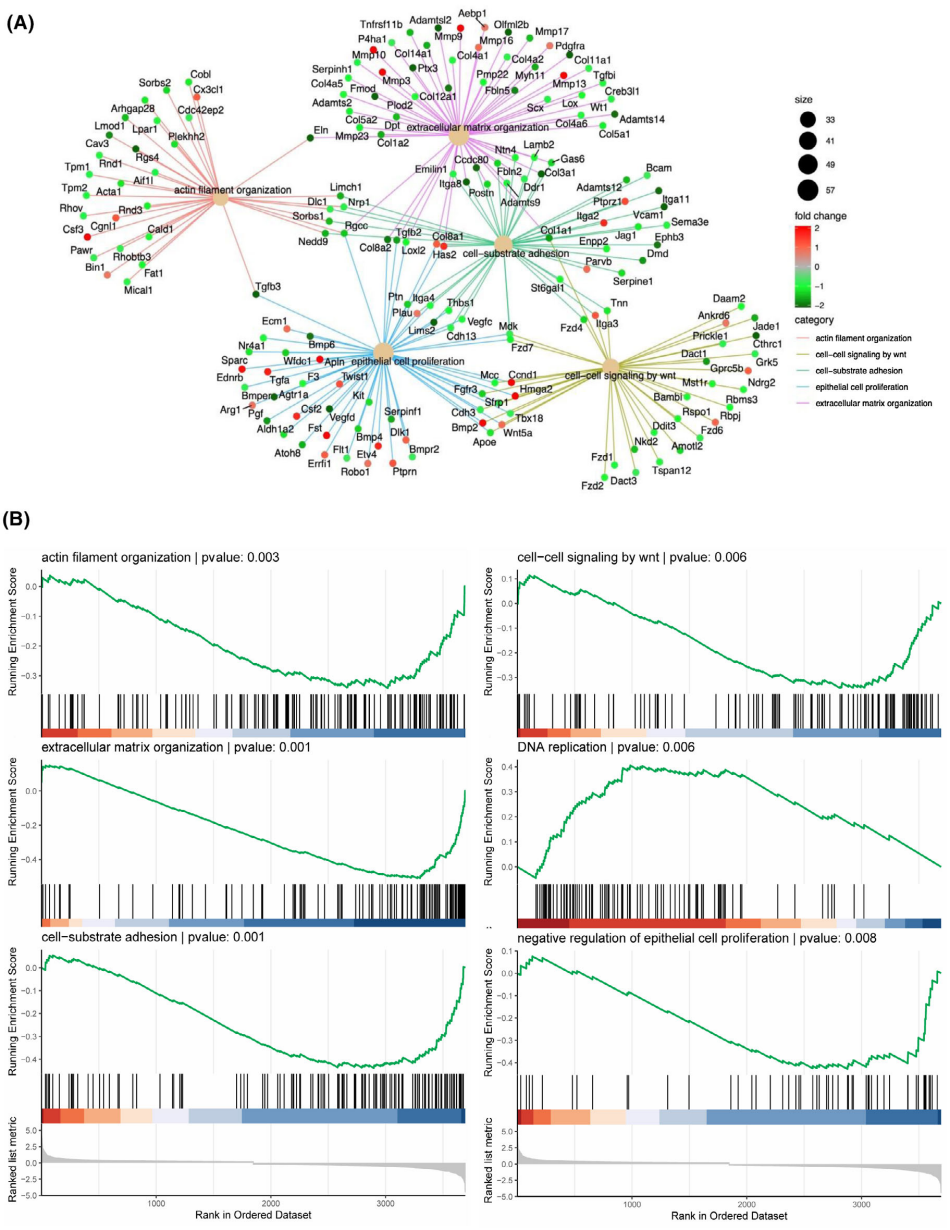
Enriched functions and genes related to PFs proliferation and activation. (A) The Gene‐Concept Network of DEGs functional enrichment in BP. (B) The analysis of the candidate functions by the GSEA method.

The principal functions enriched in the BP are selected and their related genes are presented (Fig. 5A). These enriched functions contain many fundamental genes related to fibroblast differentiation and fibrosis. The expression of most DEGs regulated actin filament organization was strongly decreased by FGF2 (Fig. 5A). In Wnt signaling pathway cluster, 23 genes were strongly decreased among the total 29 DEGs, indicating that FGF2 may play an important role in regulating the Wnt signaling pathway. At the same time, many genes related to extracellular structure organization were also decreased, including *Col3a1*, *Col12a1*, *Col1a2*, *Col14a1*, *Col4a1*, *Col4a2*, *Lamb2*, *Postn*, and *Ccdc80*, while some genes associated with matrix protein degradation (*e.g*., *Mmp3*, *Mmp10*, and *Mmp13*) were dramatically increased (Fig. 5A). Many main genes related to cell adhesion were suppressed in the network, such as *Itga11*, *Itga2*, *Itga3*, *Itg8*, and *Seprine* (Fig. 5A). These results indicate that FGF2 could function as attenuating the activation of PFs and inhibiting the expression of profibrotic genes in PFs. In this study, GSEA was applied to determine whether the biological function priori defined by a set of genes is significantly upregulated or downregulated. The enrichment scores of extracellular structure organization, cell adhesion, Wnt signaling pathway, and cell cytoskeleton organization are all negative, while the score of DNA replication is positive (Fig. 5B), suggesting FGF2 signaling can downregulate ECM organization, adhesion, and cytoskeleton organization, and upregulate DNA replication in PFs.

### Validation of regulatory roles of FGF2 in PFs

**Fig. 6:**
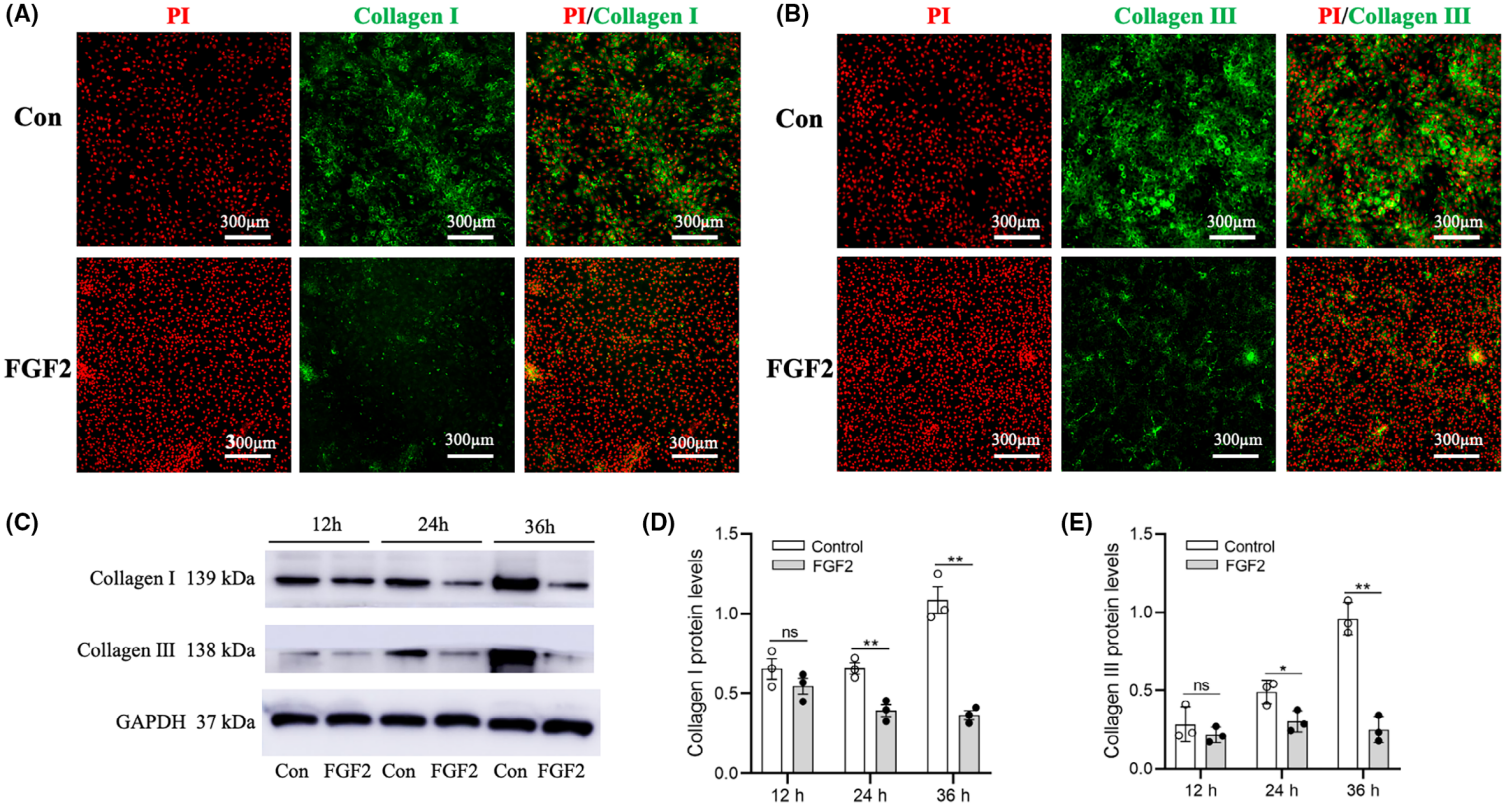
Expressions of collagen type I and type III in PFs. (A, B) Collagen type I and collagen type III expressions in PFs by immunofluorescence staining. Scale bar: 300 μm. (C–E) The protein levels of collagen type I and collagen type III were determined by western blot analysis. The data are presented as means ± SEM and analyzed using an unpaired Student's *t*‐test for statistical comparison (ns, no significant difference; *P < 0.05, **P < 0.01, *n* = 3).

**Fig. 7:**
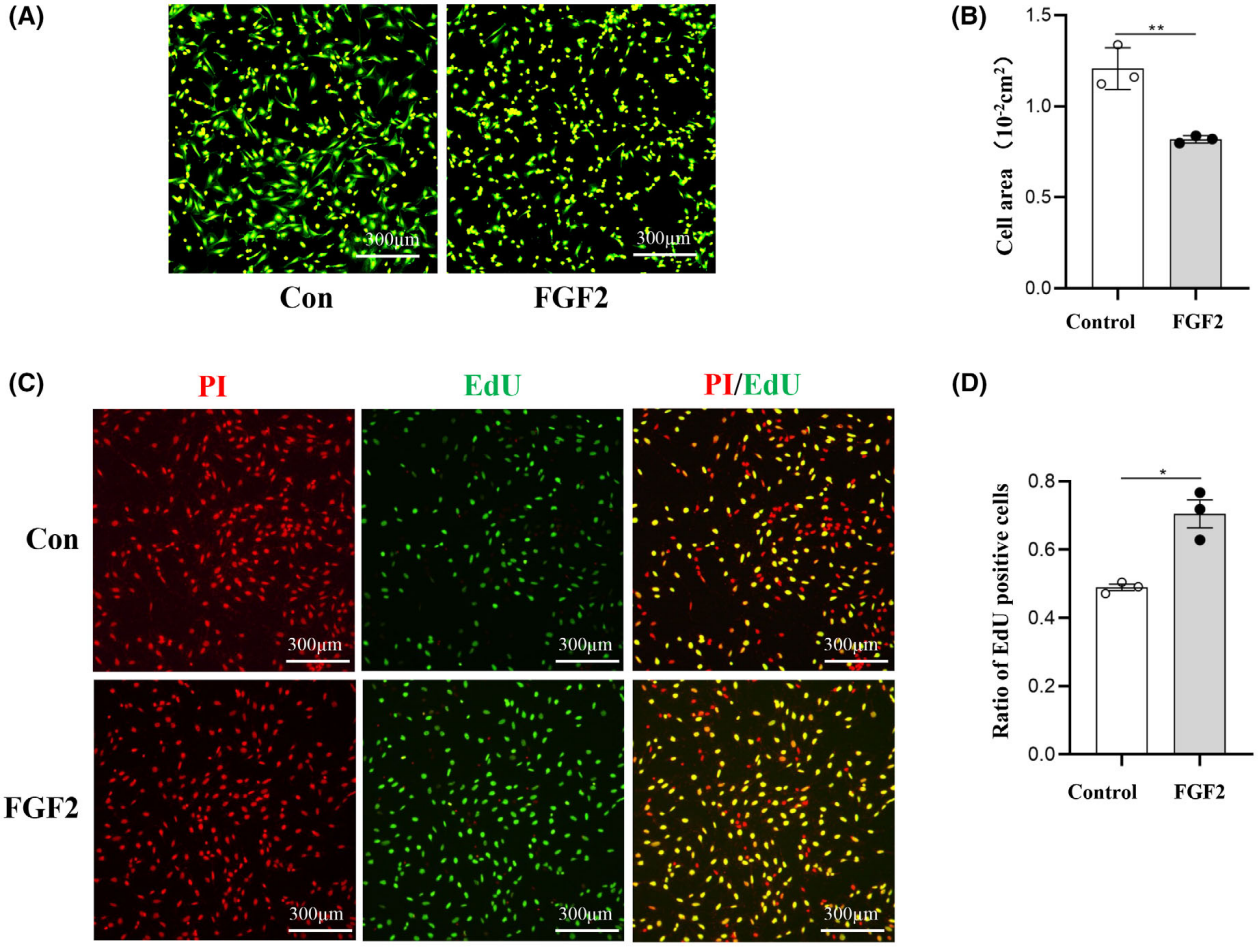
Effects of FGF2 on PFs adhesion and proliferation. (A, B) The adhesion differences between the FGF2 treatment group and the control group. Scale bar: 300 μm. (C, D) PFs proliferation was assessed by EdU. Scale bar: 300 μm. The data are presented as means ± SEM and analyzed using an unpaired Student's *t*‐test for statistical comparison (*P < 0.05, **P < 0.01, *n* = 3).

We evaluated the influence of FGF2 on collagen matrix expression, adhesion, and proliferation of PFs. After treatment with FGF2 for 48 h, the expression levels of collagen type I and III were both significantly decreased in PFs determined by immunostaining (Fig. 6A,B) and by western blot (Fig. 6C–E). To study the adhesion of PFs, the briefly trypsinized cells were stained with CFSE to measure the cell surface. The averaged cell area of the FGF2 group was significantly smaller than that in the control group, suggesting that FGF2 is capable of inhibiting cell adhesion (Fig. 7A,B). EdU assay was applied to detect the effect of FGF2 on PFs proliferation, and the ratios of positively stained cells for the FGF2 group and control group reached 70% and 57%, respectively, suggesting FGF2 promoted DNA replication in PFs (Fig. 7C,D).

**Fig. 8:**
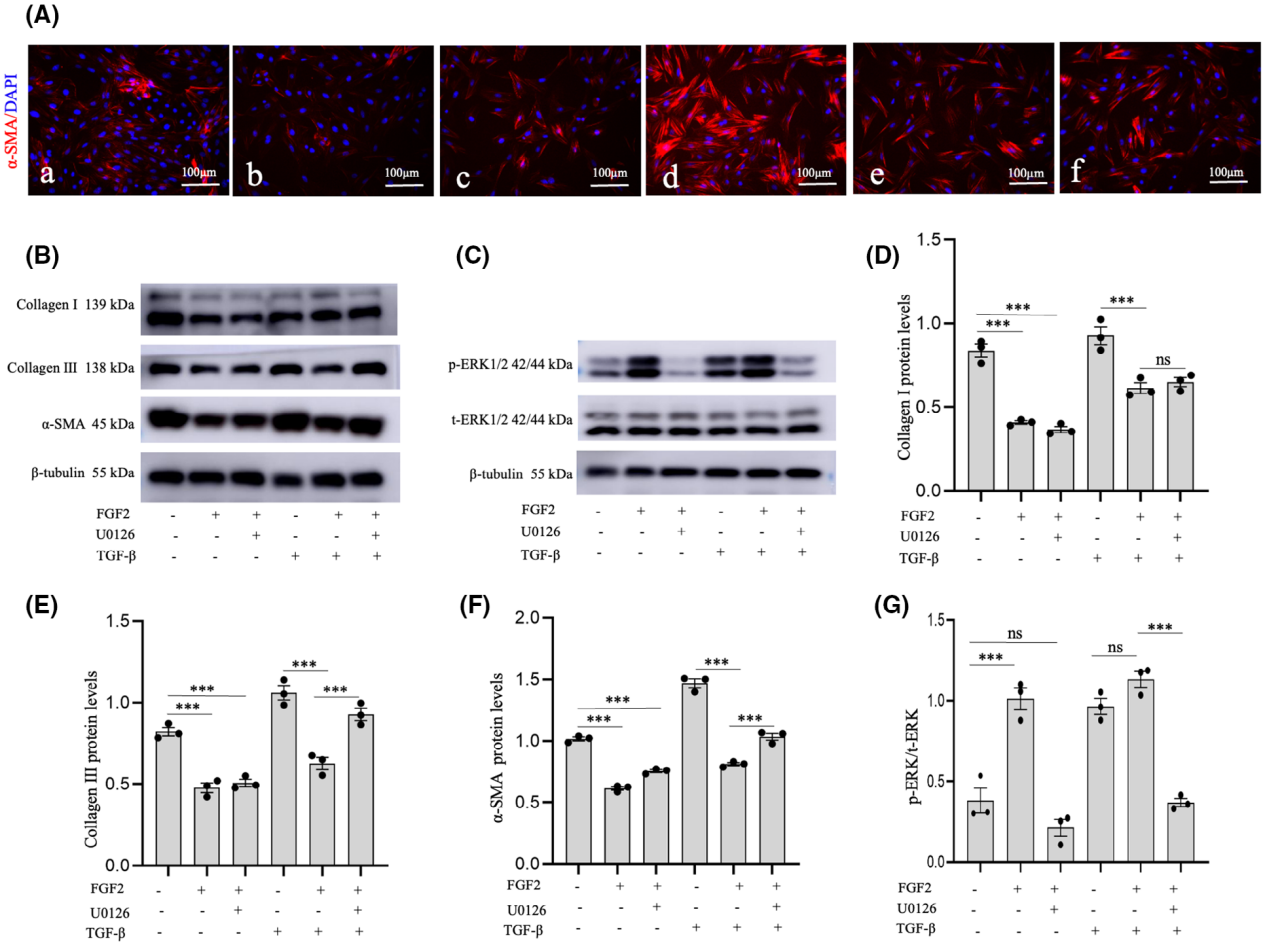
Inhibitory effect of FGF2 on PFs activation in the absence and presence of TGF‐β1 and ERK1/2 inhibitor. (A) Immunofluorescence staining of expression of α‐SMA after treatment with FGF2, U0126, or TGF‐β1 (a: control, b: treated with FGF2, c: treated with FGF2 and U0126, d: treated with TGF‐β1, e: treated with TGF‐β1 and FGF2, f: treated with TGF‐β, FGF2, and U0126). Scale bar: 100 μm. (B) Expression levels of collagen type I and III (COL I and COL III) and α‐SMA in PFs were measured by western blot. (C) Expression levels of p‐ERK1/2 and total‐ERK1/2 measured by western blot after 1 h treatment with FGF2, U0126, or TGF‐β1. (D–G) The analysis of protein expression levels of collagen type I, collagen type III, α‐SMA, and p‐ERK/total‐ERK. The data are presented as means ± SEM and analyzed using one‐way ANOVA followed by Bonferroni's multiple‐comparisons *post hoc* test for statistical comparison (ns, no significant difference; ***P < 0.001, *n* = 3).

Recombinant TGF‐β1 protein and U0126 (an ERK1/2 inhibitor) were used to determine the effect of FGF2 on PFs activation and the relevant signaling regulation. The immunofluorescence results show that TGF‐β1 (2 ng·mL^−1^) increased α‐SMA expression and stress fiber formation, while FGF2 suppressed the expression of α‐SMA induced by mechanical stress as cultured *in vitro* or by TGF‐β1 (Fig. 8A,B,F). Addition of U0126 suppressed the effect of FGF2 on regulation of α‐SMA expression. Similarly, FGF2 reduced the expression of collagen type I and III (COL I and COL III) in both the absence and presence of TGF‐β1 (Fig. 8B,D,E). Interestingly, the ERK inhibitor U0126 could only block the inhibiting effect of FGF2 on the expression of α‐SMA and collagen type III induced by TGF‐β1 (Fig. 8B,D,E). The western blot result shows that FGF2 could induce the phosphorylation of ERK1/2, and the phosphorylation level was decreased after treatment with U0126 (Fig. 8C,G). Taken together, these results suggest FGF2 can regulated PFs activation by ERK signaling, but multiple other signaling pathways may also be involved in the regulation of profibrotic gene expression.

## Discussion

Previous studies had shown that FGF2 is highly pleiotropic and its receptors are expressed on cells of multiple types [23]. The function of FGF2 signaling in the process of fibrosis is complex [24]. In this study, we shed light on the roles of FGF2 in the regulation of PFs and found that the effect of FGF2 on PFs are multifaceted, including reverting PFs differentiation into myofibroblasts, decreasing the expression of ECM, increasing matrix metalloproteinases (MMPs) expression, inhibiting cytoskeleton formation, and reducing their adhesion ability.

Fibroblasts regulate the composition of ECM by modulating the expression of collagens, laminin, fibronectin, and MMPs [25]. MMPs are proteinases that, together, can degrade ECM and numerous nonmatrix proteins [26]. In this study, we found that the expression of genes, including collagen, laminin, and elastin, was markedly downregulated after FGF2 treatment. By contrast, *Mmp3*, *Mmp10*, and *Mmp13* were strongly upregulated. Enrichment analysis revealed that the genes related to extracellular structure organization were mainly negatively regulated, suggesting FGF2 plays a key role in regulating the aberrant matrix remodeling. These results indicate that FGF2 can attenuate the formation of ECM and has the function of increasing the degradation of ECM in PFs.

It is known that a typical feature of myofibroblasts, their contractile activity depends on the expression and organization of α‐SMA [27]. In the present work, we found that α‐SMA synthesis in fibroblasts decreased dramatically after FGF2 treatment, and expression of the genes related to actin filament and actomyosin structure organization were also remarkably suppressed, indicating that FGF2 is capable of inhibiting the formation of the cytoskeleton. The cytoskeleton is pivotal for controlling cell shape and motility, and for organizing signaling complexes [28]. Additionally, associative studies have demonstrated that cytoskeletal proteins play important role in cell migration and adhesion [29]. Focal adhesions provide dynamic links between the actin cytoskeleton and ECM [30, 31], which facilitate communication between ECM and cell cytoskeleton, which is mediated through FGF2 binding integrin receptor on the cell surface [32]. RNA sequencing revealed that the expression of genes related to cell adhesion, including *Itgα*, *Itgβ*, and *Ccdc80*, were significantly reduced after FGF2 treatment. We speculate that FGF2 may be involved in modulating cellular size by restraining cytoskeleton formation and reducing cell adhesion. It is well known that there is a reciprocal link between the cell‐cycle machinery and adhesion complexes/cytoskeleton [29]. Some studies believe that the increase in cytoskeletal proteins and the enhancement of cell adhesion ability contribute to the proliferation and division of cells [33, 34, 35].However, our study revealed that the genes associated with DNA replication were significantly upregulated after FGF2 treatment. Meanwhile, the synthesis of cytoskeletal proteins and cell adhesion capacity was significantly decreased. Thus, it is likely that the coordination of DNA replication with the cytoskeleton and cell adhesion is complicated and requires further research.

It is well established that the TGF‐β signaling pathway plays a crucial role in regulating lung fibrosis [36]. FGF2 has been proposed to both facilitate and augment the fibrotic response to TGF‐β1, which is central to the pathogenesis of pulmonary [10]. In this study, we found that FGF2 significantly reduced gene expression of *Tgfb2*, and *Tgfb3* in lung fibroblasts, which was consistent with previous studies [37]. Meanwhile, FGF2 could inhibit the up‐regulation effect of TGF‐β on the expression of COL I, COL III, and α‐SMA protein, indicating that FGF2 could suppress the differentiation of PFs induced by TGF‐β. Furthermore, the genomic enrichment analysis showed that the FGF2 gene was enriched and concentrated in the Wnt pathway. Most of the genes related to the Wnt pathway were downregulated. The Wnt signaling cascade contributes to fibrotic disorders in multiple target organs including systemic sclerosis, kidney fibrosis, diabetic retinopathy, and dermal scarring [38]. Wnt target genes show high expression in alveolar epithelial type II cells in both a mouse model of pulmonary fibrosis and patients with IPF [39]. It has been confirmed that suppression of the Wnt/β‐catenin signaling could significantly alleviate bleomycin‐induced pulmonary fibrosis accompanied by reducing expression of TGF‐β1 and FGF2 *in vitro* and *in vivo* [38]. Further studies are required to precisely understand how FGF‐2 regulates lung fibrosis.

## Conclusions

In conclusion, this study sheds light on the systematical regulatory effects of FGF2 on PFs proliferation, adhesion, and differentiation, which suggests that FGF2 acts as an upstream regulator in the progress of PFs activation. The findings would provide a basis for clinical application of FGF2 in treatment of lung repair and inhibition of pulmonary fibrosis.

## Conflict of interest

The authors declare no conflict of interest.

### Peer review

The peer review history for this article is available at https://www.webofscience.com/api/gateway/wos/peer‐review/10.1002/2211‐5463.13691.

## Author contributions

XT and CS contributed to the conceptualization, methodology, software, resources, writing—original draft, and project administration. YJ and YG contributed to the methodology and writing—review and editing. YL contributed to the methodology and software. ZT and HL contributed to the methodology. XC contributed to the writing—review and editing and project administration.

## Supporting information


**Fig. S1.** Genes in MAPK signaling pathway.
**Fig. S2.** Genes in PI3K‐AKT signaling pathway.
**Fig. S3.** Genes in WNT signaling pathway.
**Fig. S4.** Genes in Focal adhesion signaling pathway.
**Table S1.** Oligonucleotide primers for qRT‐PCR in this study.
**Table S2.** Significantly up or downregulated DEGs.

## Data Availability

The original data for mRNA sequencing are available at NCBI Sequence Read Archive (BioProject ID: PRJNA833160).
