# Supplementary material for: Fibroblast growth factor 2 acts as an upstream regulator of inhibition of pulmonary fibroblast activation
Source: FEBS Open Bio. 2023 Aug 17;13(10):1895–909. doi: 10.1002/2211-5463.13691 (PMC10549223; doi:10.1002/2211-5463.13691)

Supplementary Material to

**Fibroblast growth factor 2 acts as an upstream regulator in the inhibition of pulmonary fibroblasts activation**

**Table S1.** Oligonucleotide primers for qRT-PCR in this study

| Primer | Oligonucleotide Sequences (5'-3') |
| --- | --- |
| cola1F | GTACATCAGCCCAAACCCCA |
| cola1R | CAGGATCGGAACCTTCGCTT |
| colIIIa1F | GGCCTCCCAGAACATTACATACC |
| colIIIa1R | CATGGCCTTGCGTGTTTG |
| ElnF | AAACTGCCCTATGGAGTGGC |
| ElnR | TCCAGCACCATACTTCGCTG |
| ItgblF | TTGATCCAACTGGCGACTGG |
| ItgblR | TGGGTGCTCACACTTCTTCC |
| Itga1F | AGCCTGCTGATCGTCAAGTC |
| Itga1R | TCCTGGTGTTGTACGCACTG |
| Acta2F | CATCACCAACTGGGACGACA |
| Acta2R | TCCGTTAGCAAGGTCGGATG |
| Tpm1F | TCACTGCAAAAGAAACTCAAGG |
| Tpm1R | GTCTGTTCAGAGATGCTACGTC |
| Tpm2F | GAAGGATGAGGAGAAGATGGAG |
| Tpm2R | GTATTTGCGGTCTGAGTCCTC |
| Mmp3F | CCAGTCTACAAGTCCTCCACAG |
| Mmp3R | TCAGGGTCCAGAGAGTTAGATTTG |
| Tgfb2F | CCATCCCGCCCACTTTCTAC |
| Tgfb2R | CAATCCGTTGTTCAGCCACTC |
| Tgfb3F | CCAGTCCAATTCCTCCTCCTCCTC |
| Tgfb3R | CTTGCTGCCTTCGTGCTGTCTC |
| Hmga1F | GAAGCAAGAACAAGGGCACAG |
| Hmga1R | CCTCTTCCTCCTTCTCCAGTTTC |
| Thbs2F | CCACCAGAACAACCAAGACAAC |
| Thbs2R | ATCATCGGAGTCACAGGCATC |
| Fgfr3F | GGCAAGCACAAGAACATCATTAAC |
| Fgfr3R | GGAGGAACTCTCGCAGGTTG |
| LoxF | CCTACTACATCCAGGCATCCAC |
| LoxR | ATCCGCCCTATATGCTGAACTG |
| Nid1F | AGAACACTGACCTACACTCCTATG |
| Nid1R | ATCCGATGATGCCTCCAATGG |
| β-actinF | TGAGCTGCGTTTTACACCCT |
| β-actinR | ATGTTTGCTCCAACCAACTGC |
| GAPDHF | ACACCCACTCCTCCACCTTT |
| GAPDHR | TTACTCCTTGGAGGCCATGT |
| Ccnd1F | CTACCGCACAACGCACTTTC |
| Ccnd1R | GCAGGCTTGACTCCAGAAGG |
| Ccdc80F | GGATGACGAGGACTTGGTAGAC |
| Ccdc80R | AACACGGACTTCATTGCTATTGG |
| Myl9F | ATGCCTTTGCCTGCTTTGATG |
| Myl9R | TCTCGTCCACCTCCTCATCC |
| Nid1F | AGAACACTGACCTACACTCCTATG |
| Nid1R | ATCCGATGATGCCTCCAATGG |
| RgccF | CCCTGGCAAAATCGGCTACT |
| RgccR | TGGTGCAGGCAGGTAAACAA |
| TaglnF | TGGCTGAAGAATGGCGTGAT |
| TaglnR | TCCATCGTTCTTGGTCACGG |
| Gas6F | TAGAAGTCGTGGCTCGCATT |
| Gas6R | GTACCATCCACTTCCAGGGTG |
| CtgfF | GAGTGGAGCGCCTGTTCTAA |
| CtgfR | TAATAAGAAGGGCGGCGAGAC |
| Timp3F | AGACAGACGCCAGAGTCTCC |
| Timp3R | AAGTCTGTCCGGGTAGGAGG |

**Table S2.** The significantly up or downregulated DEGs

| **gene_id** | **baseMean** | **log2FoldChange** | **padj** | **GeneSymbol** |
| --- | --- | --- | --- | --- |
| ENSRNOG00000003897 | 155366.941 | -1.7109584 | 4.00E-95 | Col1a1 |
| ENSRNOG00000045829 | 89741.5353 | -1.0108737 | 7.57E-39 | Thbs1 |
| ENSRNOG00000015036 | 77440.0618 | -1.1063118 | 8.00E-106 | Ctgf |
| ENSRNOG00000058645 | 75011.5061 | -1.2864022 | 8.17E-93 | Tnc |
| ENSRNOG00000001414 | 61007.8015 | -1.275076 | 5.78E-151 | Serpine1 |
| ENSRNOG00000017628 | 55308.7867 | -0.8321238 | 7.64E-54 | Tagln |
| ENSRNOG00000058039 | 53011.8724 | -1.825028 | 3.99E-190 | Acta2 |
| ENSRNOG00000003357 | 49704.9735 | -2.4404819 | 6.97E-257 | Col3a1 |
| ENSRNOG00000016831 | 49544.4917 | -0.8704722 | 3.25E-39 | Serpinh1 |
| ENSRNOG00000012840 | 44503.7577 | -1.2091408 | 1.17E-124 | Sparc |
| ENSRNOG00000016281 | 39690.8281 | -0.9328774 | 8.14E-61 | Col4a1 |
| ENSRNOG00000008749 | 26375.0627 | -1.1286736 | 3.83E-78 | Col5a1 |
| ENSRNOG00000014426 | 25561.4535 | -1.0648165 | 1.83E-132 | Lox |
| ENSRNOG00000003736 | 25107.4348 | -1.0979554 | 8.09E-136 | Col5a2 |
| ENSRNOG00000010233 | 23742.8074 | -0.9898988 | 1.69E-43 | Cald1 |
| ENSRNOG00000018184 | 23071.2751 | -0.8791112 | 1.26E-94 | Tpm1 |
| ENSRNOG00000023972 | 19565.493 | -0.8560274 | 2.69E-50 | Col4a2 |
| ENSRNOG00000007302 | 18005.5379 | -1.6218089 | 1.62E-115 | Fbn1 |
| ENSRNOG00000014350 | 17393.0664 | -0.8201137 | 1.53E-47 | Cyr61 |
| ENSRNOG00000058470 | 16822.3407 | -1.950725 | 9.87E-248 | Col12a1 |
| ENSRNOG00000012094 | 15507.2773 | -1.2256637 | 9.28E-90 | Ltbp2 |
| ENSRNOG00000016731 | 14530.446 | -0.9708069 | 1.55E-98 | Tpm2 |
| ENSRNOG00000002746 | 14007.2913 | -1.0255517 | 7.00E-131 | Fstl1 |
| ENSRNOG00000001469 | 13673.3181 | -3.0223355 | 5.12E-158 | Eln |
| ENSRNOG00000018824 | 12838.3999 | -0.9699086 | 3.88E-62 | Slc7a5 |
| ENSRNOG00000007338 | 10128.2863 | -1.3333162 | 3.04E-111 | Fbln2 |
| ENSRNOG00000029401 | 9375.76783 | -1.5482804 | 3.30E-159 | Actg2 |
| ENSRNOG00000030954 | 9103.12154 | -1.0886846 | 4.64E-70 | Fat1 |
| ENSRNOG00000011800 | 8831.88552 | -1.0225146 | 2.32E-87 | F3 |
| ENSRNOG00000021437 | 8817.23788 | -0.8723426 | 7.49E-07 | AABR07073181.1 |
| ENSRNOG00000010529 | 8251.80171 | -2.6414252 | 1.47E-116 | Thbs2 |
| ENSRNOG00000030183 | 8148.24583 | -1.0205577 | 3.97E-77 | Plod2 |
| ENSRNOG00000048043 | 7830.47069 | -1.5923419 | 1.53E-268 | F2r |
| ENSRNOG00000009867 | 7755.62995 | -2.8909664 | 0 | Tgfb3 |
| ENSRNOG00000018487 | 7440.59983 | -0.8371967 | 5.24E-55 | Slc3a2 |
| ENSRNOG00000018836 | 7389.85995 | -1.878044 | 2.73E-238 | RGD1559896 |
| ENSRNOG00000014182 | 6995.14656 | -1.2075273 | 2.21E-21 | Tns1 |
| ENSRNOG00000002215 | 6811.26453 | -1.4805586 | 1.53E-231 | Mylk |
| ENSRNOG00000008246 | 6734.26319 | -0.8825269 | 1.10E-41 | Emilin1 |
| ENSRNOG00000020246 | 6719.13886 | -1.1873577 | 4.49E-119 | Myl9 |
| ENSRNOG00000050655 | 6100.52582 | -0.7515665 | 1.32E-58 | P4ha1 |
| ENSRNOG00000025001 | 5950.53243 | -0.8727695 | 7.72E-78 | Pcolce |
| ENSRNOG00000001607 | 5782.64567 | -2.5891376 | 0 | Adamts1 |
| ENSRNOG00000057880 | 5468.93097 | -1.6332213 | 4.30E-264 | Myh11 |
| ENSRNOG00000016758 | 5461.0896 | -1.0653203 | 1.68E-58 | Loxl2 |
| ENSRNOG00000023546 | 5375.03267 | -1.2809386 | 1.96E-76 | Hspb1 |
| ENSRNOG00000019810 | 5279.8141 | -0.9468852 | 7.04E-46 | Des |
| ENSRNOG00000008487 | 5276.8801 | -1.2863559 | 1.21E-128 | Amotl2 |
| ENSRNOG00000033663 | 5081.65672 | -0.8950906 | 6.28E-76 | P4ha2 |
| ENSRNOG00000014835 | 4917.31233 | -1.9059672 | 3.32E-244 | Il1rl1 |
| ENSRNOG00000002052 | 4916.48607 | -2.0568527 | 0 | Ccdc80 |
| ENSRNOG00000046313 | 4862.9922 | -0.870485 | 9.88E-39 | Basp1 |
| ENSRNOG00000012956 | 4661.47521 | -0.931267 | 9.92E-35 | Tgm2 |
| ENSRNOG00000021259 | 4453.05002 | -1.031216 | 5.33E-98 | Prnp |
| ENSRNOG00000008061 | 4200.99216 | -1.6112807 | 4.47E-218 | Nuak1 |
| ENSRNOG00000010107 | 4104.40711 | -1.049336 | 2.05E-65 | AABR07025295.1 |
| ENSRNOG00000001030 | 4056.21488 | -1.3786003 | 7.38E-177 | Tsc22d1 |
| ENSRNOG00000000875 | 3926.92701 | -1.5989001 | 2.67E-197 | Fhl1 |
| ENSRNOG00000027736 | 3720.27937 | -1.7662147 | 3.95E-245 | Cnn1 |
| ENSRNOG00000047768 | 3596.26415 | -1.06856 | 6.50E-73 | Lamb2 |
| ENSRNOG00000011951 | 3453.26875 | -0.9326377 | 2.66E-63 | Plk2 |
| ENSRNOG00000011292 | 3442.3173 | -1.6226109 | 1.07E-16 | Col1a2 |
| ENSRNOG00000028774 | 3281.15081 | -1.082442 | 9.71E-85 | Vgll3 |
| ENSRNOG00000012442 | 3133.56128 | -4.4765037 | 6.04E-78 | Cemip |
| ENSRNOG00000057556 | 3130.87334 | -1.9096195 | 4.62E-190 | Pdzrn3 |
| ENSRNOG00000002097 | 3074.4793 | -2.2260049 | 0 | Rasl11b |
| ENSRNOG00000021084 | 3032.41875 | -0.7793184 | 3.61E-51 | AABR07006310.1 |
| ENSRNOG00000004303 | 2977.42393 | -1.3639951 | 1.45E-94 | Timp3 |
| ENSRNOG00000005413 | 2953.32392 | -0.9792077 | 1.61E-52 | Creb3l1 |
| ENSRNOG00000016242 | 2859.20233 | -0.9427336 | 1.18E-39 | Fzd1 |
| ENSRNOG00000020456 | 2783.5557 | -0.9220971 | 8.68E-43 | Nucb2 |
| ENSRNOG00000016166 | 2770.26897 | -0.7724494 | 2.58E-45 | Pdlim1 |
| ENSRNOG00000005248 | 2662.24571 | -0.9653486 | 1.82E-46 | Slc1a4 |
| ENSRNOG00000003120 | 2621.60916 | -0.8835768 | 3.79E-48 | Prelp |
| ENSRNOG00000061484 | 2606.90386 | -0.9994302 | 1.03E-42 | Adamts2 |
| ENSRNOG00000008659 | 2600.04898 | -0.846452 | 6.69E-42 | Arhgap21 |
| ENSRNOG00000017118 | 2599.61716 | -1.2737806 | 4.56E-100 | P4ha3 |
| ENSRNOG00000003338 | 2523.41821 | -1.0480513 | 5.15E-53 | Pmp22 |
| ENSRNOG00000016538 | 2518.02769 | -2.5645291 | 0 | Itga8 |
| ENSRNOG00000031890 | 2499.99626 | -0.8755715 | 6.09E-37 | Ncam1 |
| ENSRNOG00000024503 | 2363.2556 | -1.2777219 | 6.15E-91 | Nbas |
| ENSRNOG00000014230 | 2357.25892 | -0.880032 | 1.25E-53 | Map1a |
| ENSRNOG00000003183 | 2297.21648 | -2.1071781 | 2.60E-178 | Fmod |
| ENSRNOG00000010780 | 2284.31414 | -0.8019438 | 4.50E-36 | Dlc1 |
| ENSRNOG00000015986 | 2209.52196 | -1.5717968 | 1.39E-156 | Rassf8 |
| ENSRNOG00000000824 | 2192.17442 | -0.8004877 | 3.68E-46 | Dse |
| ENSRNOG00000027030 | 2190.47552 | -1.5213328 | 4.58E-136 | Adm |
| ENSRNOG00000014524 | 2154.83929 | -1.5270645 | 4.72E-141 | S1pr3 |
| ENSRNOG00000017484 | 2145.95736 | -1.5506577 | 1.35E-26 | Gja5 |
| ENSRNOG00000002413 | 2112.13261 | -1.2041137 | 3.21E-89 | Gpc4 |
| ENSRNOG00000014683 | 2048.15549 | -1.3154973 | 2.23E-111 | Il1rl2 |
| ENSRNOG00000002418 | 2035.13919 | -1.689387 | 1.54E-121 | Tgfb2 |
| ENSRNOG00000015357 | 1999.65392 | -0.8179897 | 2.48E-45 | Bmper |
| ENSRNOG00000005690 | 1998.75456 | -0.9968856 | 1.29E-55 | Lmcd1 |
| ENSRNOG00000028274 | 1993.19095 | -0.93422 | 1.39E-52 | Myrf |
| ENSRNOG00000000940 | 1954.69401 | -0.7662852 | 1.59E-38 | Flt1 |
| ENSRNOG00000021962 | 1920.43312 | -1.0614599 | 1.39E-37 | Fzd2 |
| ENSRNOG00000032660 | 1909.41999 | -0.8108523 | 1.58E-39 | Adgrl2 |
| ENSRNOG00000013090 | 1901.66268 | -1.9390409 | 1.76E-191 | Gadd45g |
| ENSRNOG00000017307 | 1836.27708 | -1.8370655 | 2.75E-192 | Prss23 |
| ENSRNOG00000022196 | 1814.1821 | -0.7542607 | 7.55E-28 | Bmpr2 |
| ENSRNOG00000007078 | 1782.15086 | -1.3201382 | 1.10E-71 | Wisp1 |
| ENSRNOG00000018454 | 1780.21233 | -1.209123 | 3.78E-16 | Apoe |
| ENSRNOG00000010183 | 1756.22717 | -1.645718 | 1.03E-127 | Fam198b |
| ENSRNOG00000028627 | 1748.39143 | -2.7223669 | 1.13E-255 | Hmcn1 |
| ENSRNOG00000003977 | 1738.58651 | -1.2820207 | 1.15E-77 | Dusp1 |
| ENSRNOG00000008245 | 1723.91362 | -0.7935121 | 2.45E-37 | AABR07054614.1 |
| ENSRNOG00000014548 | 1719.21769 | -1.5726107 | 1.60E-102 | Nedd9 |
| ENSRNOG00000018865 | 1710.79604 | -1.5202808 | 2.54E-93 | Adamts12 |
| ENSRNOG00000007377 | 1697.41932 | -1.9194977 | 5.50E-163 | Slit3 |
| ENSRNOG00000026415 | 1697.06106 | -1.573549 | 1.27E-123 | Col14a1 |
| ENSRNOG00000033110 | 1694.86625 | -2.0694017 | 2.32E-161 | Svep1 |
| ENSRNOG00000009980 | 1684.10546 | -0.8404302 | 5.93E-32 | Plpp1 |
| ENSRNOG00000013934 | 1665.63536 | -1.1907912 | 2.12E-74 | St5 |
| ENSRNOG00000017208 | 1639.109 | -1.0557598 | 1.41E-48 | Cspg4 |
| ENSRNOG00000022772 | 1617.46589 | -0.8386737 | 1.38E-38 | Prickle1 |
| ENSRNOG00000056219 | 1603.90917 | -1.645313 | 6.64E-76 | Olr1 |
| ENSRNOG00000001979 | 1564.29375 | -1.2130863 | 2.61E-81 | Rcan1 |
| ENSRNOG00000012280 | 1556.50303 | -2.2379007 | 1.43E-235 | Ptx3 |
| ENSRNOG00000027491 | 1528.03938 | -1.8012166 | 6.58E-168 | Vldlr |
| ENSRNOG00000012563 | 1522.33723 | -0.8034271 | 2.61E-19 | Arhgap29 |
| ENSRNOG00000025394 | 1509.65091 | -1.0297163 | 1.59E-45 | Tanc1 |
| ENSRNOG00000004345 | 1493.90267 | -0.9276312 | 5.82E-46 | Daam1 |
| ENSRNOG00000012660 | 1493.07446 | -1.2998373 | 3.63E-79 | Postn |
| ENSRNOG00000013717 | 1469.67329 | -1.9742173 | 6.38E-148 | Bmp6 |
| ENSRNOG00000009951 | 1466.47658 | -0.8636825 | 4.25E-30 | Aif1l |
| ENSRNOG00000057125 | 1449.54382 | -0.8245408 | 1.13E-38 | Ddr1 |
| ENSRNOG00000000635 | 1428.44557 | -0.9667933 | 6.11E-43 | Arid5b |
| ENSRNOG00000012216 | 1421.92389 | -0.8561187 | 1.32E-26 | Tgfbi |
| ENSRNOG00000018126 | 1369.41357 | -1.0610327 | 1.64E-52 | Abca1 |
| ENSRNOG00000007090 | 1362.85707 | -1.2439504 | 1.69E-67 | Cacna1c |
| ENSRNOG00000050539 | 1362.03476 | -1.8616375 | 8.94E-133 | Fbln5 |
| ENSRNOG00000018225 | 1344.00148 | -1.0948698 | 1.19E-61 | Tp53inp2 |
| ENSRNOG00000010666 | 1338.49156 | -3.6813538 | 0 | Wisp2 |
| ENSRNOG00000000700 | 1333.9899 | -0.9993154 | 6.40E-35 | Tmem119 |
| ENSRNOG00000060603 | 1330.66721 | -1.0382197 | 1.01E-55 | Nhsl1 |
| ENSRNOG00000026060 | 1325.056 | -2.1736114 | 1.97E-138 | Arsi |
| ENSRNOG00000009311 | 1301.25274 | -1.0750654 | 1.62E-42 | Fstl3 |
| ENSRNOG00000007002 | 1265.16349 | -0.9996671 | 8.78E-53 | Lif |
| ENSRNOG00000032590 | 1261.55775 | -0.9873148 | 1.13E-30 | Ttc28 |
| ENSRNOG00000008465 | 1234.92068 | -0.8813783 | 1.34E-28 | Tmem176b |
| ENSRNOG00000015406 | 1231.11442 | -2.4173462 | 1.23E-224 | Pgm5 |
| ENSRNOG00000004624 | 1230.9652 | -1.1493686 | 1.31E-34 | Rnd3 |
| ENSRNOG00000001706 | 1217.35161 | -0.8327075 | 8.97E-31 | Kalrn |
| ENSRNOG00000025691 | 1214.92558 | -0.8740084 | 1.92E-33 | Pla2g7 |
| ENSRNOG00000018233 | 1185.7188 | -1.3044532 | 9.06E-56 | Gas6 |
| ENSRNOG00000014791 | 1184.8987 | -2.0094854 | 4.53E-175 | Peg3 |
| ENSRNOG00000023013 | 1170.94915 | -0.915872 | 2.16E-34 | 43893 |
| ENSRNOG00000010524 | 1169.94449 | -2.416399 | 7.06E-236 | Cryab |
| ENSRNOG00000032339 | 1165.56047 | -1.1071735 | 1.03E-55 | Rasl12 |
| ENSRNOG00000004279 | 1138.58263 | -0.7642105 | 1.51E-21 | Adgra3 |
| ENSRNOG00000021243 | 1127.59575 | -1.4364486 | 2.23E-56 | Siglec1 |
| ENSRNOG00000006833 | 1125.1263 | -0.759269 | 0.00024296 | Rb1cc1 |
| ENSRNOG00000000307 | 1122.8393 | -0.853542 | 1.20E-32 | Mical1 |
| ENSRNOG00000023257 | 1118.72525 | -0.9046451 | 2.46E-27 | Adamts9 |
| ENSRNOG00000025895 | 1118.24986 | -2.4017698 | 7.34E-216 | Cavin2 |
| ENSRNOG00000055049 | 1114.71534 | -1.2466156 | 2.00E-66 | Aldh1a2 |
| ENSRNOG00000013742 | 1113.52255 | -1.2934366 | 3.80E-52 | Large1 |
| ENSRNOG00000014029 | 1103.20369 | -0.8359978 | 7.62E-30 | Klhl13 |
| ENSRNOG00000005464 | 1086.10226 | -1.322338 | 4.63E-57 | Lgalsl |
| ENSRNOG00000057078 | 1084.30133 | -0.890668 | 3.79E-27 | Ddit4 |
| ENSRNOG00000018951 | 1083.34182 | -1.0478556 | 1.37E-39 | Col4a5 |
| ENSRNOG00000001828 | 1080.0784 | -1.0454185 | 1.26E-46 | Stk38l |
| ENSRNOG00000008336 | 1054.75824 | -1.5304551 | 1.19E-102 | Tnfrsf11b |
| ENSRNOG00000046254 | 1054.70359 | -1.0826911 | 6.59E-44 | Adgre1 |
| ENSRNOG00000014361 | 1051.33983 | -1.3372536 | 3.08E-09 | Edn1 |
| ENSRNOG00000028335 | 1047.09112 | -1.0435417 | 1.22E-36 | Fat4 |
| ENSRNOG00000014320 | 1043.4955 | -0.884528 | 8.64E-05 | Inhba |
| ENSRNOG00000030880 | 1042.66351 | -1.3276319 | 1.39E-70 | Hs6st2 |
| ENSRNOG00000019822 | 1024.79009 | -1.3900299 | 2.00E-61 | Gadd45b |
| ENSRNOG00000015505 | 999.9481 | -2.0714634 | 2.08E-157 | Mfap5 |
| ENSRNOG00000003031 | 996.321905 | -1.3467105 | 6.43E-65 | Atp2b4 |
| ENSRNOG00000017164 | 991.516023 | -2.7786822 | 1.44E-256 | Afap1l2 |
| ENSRNOG00000016119 | 985.261107 | -1.1131862 | 3.92E-42 | Fzd7 |
| ENSRNOG00000001227 | 984.95261 | -1.6213652 | 1.81E-72 | Adarb1 |
| ENSRNOG00000010389 | 954.473136 | -1.0250619 | 2.55E-30 | Ndrg2 |
| ENSRNOG00000051548 | 950.841596 | -1.4801311 | 2.46E-78 | Lmod1 |
| ENSRNOG00000000142 | 950.833469 | -1.092083 | 3.62E-48 | Plxdc2 |
| ENSRNOG00000039666 | 944.946592 | -1.4419054 | 2.30E-76 | Srpx |
| ENSRNOG00000031801 | 940.325535 | -1.9571106 | 6.07E-133 | Ephb3 |
| ENSRNOG00000016818 | 933.70484 | -1.3656991 | 1.54E-51 | Fgfr3 |
| ENSRNOG00000030715 | 933.683409 | -1.1932266 | 6.68E-39 | Cfh |
| ENSRNOG00000020129 | 925.017553 | -0.8202797 | 3.64E-27 | Cdh3 |
| ENSRNOG00000028910 | 920.388141 | -1.2320374 | 5.05E-41 | Ccdc9b |
| ENSRNOG00000014066 | 918.39409 | -1.6054529 | 1.16E-69 | Jade1 |
| ENSRNOG00000042960 | 916.144134 | -1.4080945 | 1.42E-16 | Rgcc |
| ENSRNOG00000007613 | 890.822223 | -1.3711243 | 1.12E-65 | C1qtnf5 |
| ENSRNOG00000015085 | 888.201319 | -2.5859745 | 1.61E-173 | Dmpk |
| ENSRNOG00000019622 | 881.539543 | -1.5310354 | 6.28E-70 | Ackr3 |
| ENSRNOG00000009636 | 873.676013 | -1.7733268 | 7.99E-96 | Scrn1 |
| ENSRNOG00000003172 | 872.943256 | -1.5968374 | 2.72E-79 | Serpinf1 |
| ENSRNOG00000017500 | 870.617125 | -1.1721194 | 2.27E-36 | Mtss1l |
| ENSRNOG00000025443 | 867.288525 | -0.77253 | 6.33E-19 | Map1lc3a |
| ENSRNOG00000007461 | 865.417664 | -0.9333085 | 3.33E-22 | Klhl41 |
| ENSRNOG00000007345 | 860.424262 | -1.0179265 | 1.21E-35 | Amot |
| ENSRNOG00000010744 | 860.129322 | -0.8468635 | 1.62E-27 | Nrp1 |
| ENSRNOG00000014746 | 856.826733 | -0.8399056 | 3.32E-22 | Dzip1l |
| ENSRNOG00000015880 | 840.663249 | -1.9246418 | 1.09E-107 | Dpep1 |
| ENSRNOG00000032989 | 840.567442 | -1.0260846 | 2.55E-35 | Lrrn4 |
| ENSRNOG00000012802 | 837.779299 | -0.9489075 | 1.58E-26 | Tenm3 |
| ENSRNOG00000007326 | 832.927452 | -0.7731028 | 5.82E-19 | Prepl |
| ENSRNOG00000015658 | 827.879002 | -1.5064539 | 1.99E-66 | Sorbs1 |
| ENSRNOG00000051440 | 815.394921 | -1.5467357 | 2.36E-78 | Ppp1r12b |
| ENSRNOG00000017065 | 792.516097 | -1.1858301 | 1.67E-35 | Arhgap28 |
| ENSRNOG00000019484 | 790.932718 | -1.0053514 | 1.84E-28 | Slc6a9 |
| ENSRNOG00000020676 | 789.246894 | -1.0148522 | 1.55E-28 | Ppp1r14a |
| ENSRNOG00000054978 | 783.118787 | -0.760574 | 7.12E-22 | Hist1h1c |
| ENSRNOG00000029399 | 778.234259 | -0.758496 | 4.18E-18 | Bcam |
| ENSRNOG00000000563 | 777.287435 | -2.0212743 | 2.57E-114 | Adamts14 |
| ENSRNOG00000012414 | 773.137429 | -0.8189813 | 8.82E-22 | Rhobtb3 |
| ENSRNOG00000059857 | 773.027278 | -0.9314846 | 2.53E-28 | Rnd1 |
| ENSRNOG00000018220 | 767.488483 | -0.8353103 | 1.01E-24 | Pde4dip |
| ENSRNOG00000007319 | 756.064591 | -1.3597533 | 1.26E-38 | Trib3 |
| ENSRNOG00000012512 | 754.01964 | -0.9300676 | 1.35E-06 | Nexn |
| ENSRNOG00000005917 | 747.161825 | -0.8123844 | 4.94E-21 | Pawr |
| ENSRNOG00000018598 | 739.107794 | -1.4151749 | 1.51E-52 | Ankrd1 |
| ENSRNOG00000008586 | 729.458484 | -1.5065301 | 1.07E-65 | Aldh1l2 |
| ENSRNOG00000011439 | 728.45993 | -0.8264445 | 1.18E-21 | Grk5 |
| ENSRNOG00000051257 | 712.685365 | -0.8068787 | 2.18E-05 | AABR07039356.2 |
| ENSRNOG00000045992 | 710.731834 | -0.8858519 | 7.91E-26 | Tlr8 |
| ENSRNOG00000002318 | 704.934595 | -1.5944586 | 1.50E-75 | Limch1 |
| ENSRNOG00000016062 | 699.985668 | -0.9101533 | 2.20E-17 | Snta1 |
| ENSRNOG00000012879 | 678.04214 | -1.8517225 | 1.96E-85 | Fabp3 |
| ENSRNOG00000004861 | 677.207276 | -0.9715587 | 2.57E-30 | Itga4 |
| ENSRNOG00000018052 | 676.163105 | -1.126484 | 2.68E-36 | Cnksr3 |
| ENSRNOG00000060775 | 673.64971 | -1.1938451 | 3.27E-35 | Lmo7 |
| ENSRNOG00000014333 | 669.291758 | -2.5617671 | 2.45E-166 | Vcam1 |
| ENSRNOG00000013928 | 657.693047 | -0.843881 | 5.90E-18 | Dsp |
| ENSRNOG00000039902 | 653.02236 | -2.499307 | 4.00E-120 | Lbh |
| ENSRNOG00000011913 | 639.350428 | -1.0790399 | 4.88E-29 | Cp |
| ENSRNOG00000005650 | 638.581905 | -1.7396109 | 4.44E-87 | Pgf |
| ENSRNOG00000016103 | 628.216463 | -1.4601102 | 3.84E-58 | Nkd2 |
| ENSRNOG00000000894 | 621.191276 | -1.0718577 | 4.58E-27 | Fry |
| ENSRNOG00000003018 | 620.398572 | -2.1127539 | 9.01E-109 | Olfml2b |
| ENSRNOG00000011668 | 611.564227 | -1.3414133 | 9.06E-42 | Nfil3 |
| ENSRNOG00000023433 | 606.480437 | -1.2191758 | 9.09E-43 | Gata6 |
| ENSRNOG00000023708 | 601.562078 | -0.884628 | 9.95E-19 | Tmem176a |
| ENSRNOG00000000704 | 600.313757 | -1.0056391 | 4.93E-24 | Cmklr1 |
| ENSRNOG00000009694 | 597.875954 | -1.343803 | 4.80E-47 | Bmp4 |
| ENSRNOG00000009656 | 596.959419 | -1.0739051 | 1.79E-23 | Rspo1 |
| ENSRNOG00000030210 | 595.267728 | -1.6850402 | 5.81E-65 | Fndc1 |
| ENSRNOG00000013011 | 588.660777 | -0.9847433 | 3.39E-19 | Dnajb4 |
| ENSRNOG00000010077 | 588.432884 | -1.1772481 | 2.83E-36 | Smarcd3 |
| ENSRNOG00000019850 | 587.705 | -1.2194944 | 1.20E-36 | Speg |
| ENSRNOG00000011416 | 575.96731 | -0.9690632 | 5.39E-24 | Vegfc |
| ENSRNOG00000020467 | 568.350056 | -0.9893351 | 1.10E-24 | Nrep |
| ENSRNOG00000013656 | 556.82939 | -0.9763226 | 1.76E-24 | Lpar1 |
| ENSRNOG00000032798 | 556.442845 | -0.9497082 | 1.44E-22 | Slco3a1 |
| ENSRNOG00000003772 | 556.397557 | -0.8704609 | 1.28E-17 | Csrp2 |
| ENSRNOG00000008943 | 552.146701 | -1.524974 | 1.20E-57 | Penk |
| ENSRNOG00000023148 | 545.885832 | -4.17216 | 8.88E-218 | Col11a1 |
| ENSRNOG00000016848 | 533.884771 | -1.305847 | 3.45E-43 | Fzd4 |
| ENSRNOG00000052256 | 531.022542 | -0.7519725 | 2.43E-11 | Dact3 |
| ENSRNOG00000005711 | 529.355218 | -1.326533 | 7.71E-44 | Ptprd |
| ENSRNOG00000019382 | 519.863077 | -1.0128188 | 2.06E-24 | Zbtb47 |
| ENSRNOG00000013017 | 515.273666 | -1.0728816 | 4.16E-28 | Arnt2 |
| ENSRNOG00000002229 | 514.188821 | -0.9392859 | 5.52E-19 | Adcy5 |
| ENSRNOG00000016012 | 513.487365 | -1.1356378 | 2.23E-34 | Spats2l |
| ENSRNOG00000006410 | 508.614621 | -1.996962 | 1.67E-76 | Akap5 |
| ENSRNOG00000013572 | 508.070573 | -0.8073197 | 4.37E-15 | Lxn |
| ENSRNOG00000017477 | 499.847066 | -1.1445132 | 4.05E-31 | Mmp23 |
| ENSRNOG00000014686 | 498.880318 | -2.1588074 | 2.18E-93 | Kcnd3 |
| ENSRNOG00000004578 | 494.60133 | -2.3684537 | 1.06E-101 | Cthrc1 |
| ENSRNOG00000046428 | 486.965648 | -0.8155262 | 1.47E-17 | Lrrc75b |
| ENSRNOG00000001823 | 484.489486 | -1.0800839 | 2.57E-25 | St6gal1 |
| ENSRNOG00000026186 | 479.305748 | -1.2364609 | 1.81E-36 | Syde2 |
| ENSRNOG00000005124 | 470.132941 | -1.2351647 | 2.53E-34 | Plekhh2 |
| ENSRNOG00000003134 | 469.826263 | -0.8883137 | 1.27E-19 | Slc4a4 |
| ENSRNOG00000012422 | 467.84254 | -1.0130106 | 7.87E-21 | Tnik |
| ENSRNOG00000014371 | 467.179056 | -0.8426716 | 1.43E-14 | Cdh13 |
| ENSRNOG00000042915 | 465.231299 | -0.8052162 | 2.76E-15 | Mxra7 |
| ENSRNOG00000008118 | 434.423335 | -1.3774254 | 2.44E-38 | Sync |
| ENSRNOG00000057794 | 434.365679 | -1.9351089 | 6.59E-63 | Adamts5 |
| ENSRNOG00000024433 | 433.773077 | -0.8203026 | 2.25E-12 | Fbxl7 |
| ENSRNOG00000029079 | 426.259542 | -3.1362124 | 8.17E-148 | Hspb7 |
| ENSRNOG00000000842 | 422.042494 | -1.1006136 | 3.02E-21 | Ddah2 |
| ENSRNOG00000013074 | 421.579806 | -0.8880622 | 8.47E-17 | Wt1 |
| ENSRNOG00000007202 | 419.239132 | -1.1578002 | 8.24E-19 | Sema3d |
| ENSRNOG00000004281 | 417.627124 | -1.1585037 | 1.99E-28 | Cobl |
| ENSRNOG00000010805 | 410.363125 | -1.3046896 | 3.35E-27 | Fabp4 |
| ENSRNOG00000016700 | 410.318346 | -0.9535858 | 4.26E-17 | Tcf21 |
| ENSRNOG00000000500 | 404.969908 | -1.2755703 | 3.29E-08 | Scube3 |
| ENSRNOG00000014902 | 403.476134 | -1.3740782 | 2.77E-32 | Stx11 |
| ENSRNOG00000004660 | 400.435242 | -0.8214843 | 3.86E-13 | Fzd6 |
| ENSRNOG00000033527 | 397.387473 | -1.0338672 | 4.55E-18 | Pappa1 |
| ENSRNOG00000048239 | 391.287652 | -1.2068557 | 1.55E-25 | Cxcr1 |
| ENSRNOG00000033496 | 384.341773 | -0.8619948 | 4.52E-12 | Igdcc4 |
| ENSRNOG00000018937 | 379.727517 | -1.1174108 | 1.86E-24 | Gstm2 |
| ENSRNOG00000016998 | 373.648953 | -0.913918 | 2.90E-13 | Atxn1 |
| ENSRNOG00000011526 | 373.281536 | -0.863678 | 1.71E-14 | Pcsk6 |
| ENSRNOG00000011140 | 371.293161 | -1.4933254 | 1.55E-34 | Fam213a |
| ENSRNOG00000014387 | 370.464411 | -1.3155546 | 1.29E-20 | Chac1 |
| ENSRNOG00000060687 | 365.425818 | -1.6883054 | 7.89E-51 | Slc24a3 |
| ENSRNOG00000013046 | 362.666593 | -0.8684035 | 7.07E-12 | Tram2 |
| ENSRNOG00000016208 | 361.768922 | -2.0452231 | 1.72E-68 | Setbp1 |
| ENSRNOG00000024435 | 361.228288 | -0.7842908 | 6.10E-12 | AABR07035946.1 |
| ENSRNOG00000011541 | 354.791339 | -1.6268474 | 1.35E-42 | Cygb |
| ENSRNOG00000010392 | 350.45281 | -1.3546527 | 2.77E-34 | Nrg1 |
| ENSRNOG00000016874 | 349.686316 | -0.755411 | 2.30E-10 | Zfp521 |
| ENSRNOG00000010240 | 347.795259 | -0.7768292 | 9.06E-09 | Fam46a |
| ENSRNOG00000007443 | 347.506876 | -1.1797188 | 7.07E-24 | Jag1 |
| ENSRNOG00000018770 | 345.999585 | -0.7834694 | 5.20E-12 | Pmaip1 |
| ENSRNOG00000059903 | 337.648987 | -0.9650121 | 1.70E-17 | Thbs3 |
| ENSRNOG00000010748 | 336.115633 | -0.9904383 | 2.53E-17 | Mtus1 |
| ENSRNOG00000022356 | 335.982644 | -0.8661595 | 2.82E-13 | Zc2hc1a |
| ENSRNOG00000020113 | 334.378971 | -1.5989098 | 1.94E-36 | Cnnm2 |
| ENSRNOG00000016957 | 333.706222 | -2.1663612 | 6.79E-59 | Igfbp2 |
| ENSRNOG00000018231 | 332.931007 | -0.807795 | 6.39E-11 | Nacc2 |
| ENSRNOG00000033119 | 332.167968 | -1.5938191 | 8.90E-37 | Plcb4 |
| ENSRNOG00000017414 | 330.870155 | -1.0082715 | 2.52E-17 | Irf7 |
| ENSRNOG00000015904 | 330.176617 | -1.3640162 | 2.32E-24 | Wfdc1 |
| ENSRNOG00000043167 | 325.750081 | -1.4688481 | 3.50E-30 | Itga9 |
| ENSRNOG00000018812 | 318.02721 | -1.0129886 | 2.05E-18 | Rpp25 |
| ENSRNOG00000003870 | 309.945941 | -0.7561756 | 1.01E-09 | C1qtnf2 |
| ENSRNOG00000051039 | 308.058019 | -1.1194228 | 1.65E-15 | LOC108349594 |
| ENSRNOG00000006857 | 306.694877 | -1.078992 | 2.76E-19 | AABR07060833.1 |
| ENSRNOG00000061895 | 303.698032 | -1.212796 | 3.06E-20 | Ly49si1 |
| ENSRNOG00000006966 | 303.632046 | -0.7610309 | 1.23E-10 | Nfia |
| ENSRNOG00000003587 | 303.347459 | -3.0770185 | 5.96E-108 | Vegfd |
| ENSRNOG00000006723 | 303.268058 | -2.0390394 | 9.70E-51 | Itga11 |
| ENSRNOG00000002381 | 302.51128 | -0.7672865 | 8.36E-09 | Bmp3 |
| ENSRNOG00000007367 | 299.548359 | -1.5990207 | 3.75E-37 | 44078 |
| ENSRNOG00000016037 | 297.451458 | -0.7751136 | 4.94E-07 | Mafb |
| ENSRNOG00000004964 | 294.207601 | -0.8504282 | 4.08E-11 | Erbb3 |
| ENSRNOG00000020922 | 294.007905 | -0.8486163 | 2.13E-12 | Hspb6 |
| ENSRNOG00000025625 | 292.086301 | -0.8072891 | 3.87E-10 | Rnase4 |
| ENSRNOG00000020904 | 289.946213 | -0.7725705 | 2.36E-10 | Cdc42ep2 |
| ENSRNOG00000026604 | 289.94321 | -1.1106505 | 5.87E-20 | Cercam |
| ENSRNOG00000017879 | 285.884987 | -0.9884897 | 4.28E-16 | Gab1 |
| ENSRNOG00000016021 | 282.448981 | -2.1228993 | 1.43E-59 | Lims2 |
| ENSRNOG00000031834 | 281.478982 | -2.2332498 | 1.07E-60 | Nkain4 |
| ENSRNOG00000058329 | 280.172513 | -1.0063825 | 7.15E-14 | Prrx2 |
| ENSRNOG00000021474 | 279.337455 | -0.9915577 | 4.50E-14 | Siglec5 |
| ENSRNOG00000014549 | 276.37429 | -1.3114783 | 1.08E-21 | Arhgef26 |
| ENSRNOG00000011068 | 274.486157 | -1.3511684 | 1.55E-28 | Papss2 |
| ENSRNOG00000026120 | 274.178004 | -0.9002051 | 1.38E-12 | Fam8a1 |
| ENSRNOG00000009117 | 273.597279 | -0.9417711 | 3.34E-11 | Otub2 |
| ENSRNOG00000015157 | 272.958532 | -1.3450761 | 7.17E-07 | Smtnl2 |
| ENSRNOG00000053045 | 270.69751 | -4.4987775 | 1.59E-116 | Wscd2 |
| ENSRNOG00000011696 | 270.280247 | -0.9270975 | 1.89E-13 | Lifr |
| ENSRNOG00000033528 | 268.903314 | -1.1120735 | 8.81E-17 | Tll1 |
| ENSRNOG00000042860 | 266.141353 | -1.1745445 | 2.35E-20 | Pappa2 |
| ENSRNOG00000057834 | 265.307701 | -1.9747072 | 5.19E-49 | LOC102553018 |
| ENSRNOG00000011271 | 261.49153 | -1.0082117 | 9.06E-16 | Mcc |
| ENSRNOG00000010841 | 261.181943 | -1.9270929 | 6.79E-48 | Col8a2 |
| ENSRNOG00000040350 | 258.430044 | -1.1341235 | 1.71E-14 | Mir675 |
| ENSRNOG00000021198 | 256.938079 | -0.9295761 | 1.02E-13 | LOC100910200 |
| ENSRNOG00000008015 | 252.505514 | -1.2748888 | 1.37E-23 | Fos |
| ENSRNOG00000058983 | 252.282373 | -1.0763395 | 5.64E-15 | Gata5 |
| ENSRNOG00000008445 | 250.062757 | -1.7273313 | 2.74E-38 | Dact1 |
| ENSRNOG00000059016 | 245.500329 | -0.9366618 | 2.47E-12 | Tspan12 |
| ENSRNOG00000004133 | 245.429062 | -0.863921 | 6.04E-09 | Slc7a3 |
| ENSRNOG00000016294 | 244.291991 | -1.4917553 | 4.96E-28 | Cd4 |
| ENSRNOG00000025881 | 242.939357 | -1.0399751 | 1.60E-14 | Rbms3 |
| ENSRNOG00000010224 | 242.509373 | -1.1430899 | 1.51E-18 | Rab30 |
| ENSRNOG00000017209 | 240.456533 | -1.9830473 | 1.03E-36 | Tubb3 |
| ENSRNOG00000007663 | 239.48239 | -0.8849984 | 0.01239258 | LOC100911372 |
| ENSRNOG00000000443 | 238.89076 | -0.8949621 | 3.36E-07 | LOC103689965 |
| ENSRNOG00000017786 | 237.376214 | -1.210962 | 6.32E-17 | Acta1 |
| ENSRNOG00000002947 | 235.803913 | -1.500586 | 8.33E-28 | Dpt |
| ENSRNOG00000023686 | 230.833142 | -1.6571791 | 2.58E-30 | Upk3b |
| ENSRNOG00000028856 | 230.374545 | -1.3349135 | 5.19E-19 | Pknox2 |
| ENSRNOG00000046621 | 229.617995 | -0.8700768 | 1.02E-08 | AABR07043748.1 |
| ENSRNOG00000014276 | 228.705428 | -1.1936708 | 2.71E-19 | Plce1 |
| ENSRNOG00000013391 | 223.768382 | -1.3106362 | 2.41E-20 | Sorbs2 |
| ENSRNOG00000016687 | 223.512731 | -1.6515269 | 9.20E-24 | Ssc5d |
| ENSRNOG00000036604 | 222.786096 | -1.3221036 | 1.20E-15 | Ifit2 |
| ENSRNOG00000020310 | 222.771611 | -0.7580561 | 4.87E-08 | Grik5 |
| ENSRNOG00000015052 | 221.807368 | -0.9073394 | 4.83E-10 | Star |
| ENSRNOG00000003494 | 220.620805 | -1.5120214 | 1.50E-27 | Ppfia4 |
| ENSRNOG00000006631 | 218.878097 | -0.9205031 | 1.65E-11 | Sema3e |
| ENSRNOG00000017021 | 216.833627 | -1.5459082 | 1.74E-28 | Galnt18 |
| ENSRNOG00000051756 | 214.414751 | -0.8787148 | 1.05E-06 | Zfp62 |
| ENSRNOG00000061100 | 214.075466 | -0.9003801 | 8.21E-11 | Tmem150a |
| ENSRNOG00000053875 | 213.683066 | -0.976688 | 5.51E-10 | Nacad |
| ENSRNOG00000053945 | 210.36322 | -1.0373596 | 9.62E-13 | Daam2 |
| ENSRNOG00000048433 | 209.002539 | -0.8375251 | 2.32E-07 | Tshz2 |
| ENSRNOG00000018346 | 206.529185 | -2.0909174 | 4.38E-47 | Agtr1a |
| ENSRNOG00000058522 | 206.409231 | -1.3748399 | 1.50E-23 | Fam214a |
| ENSRNOG00000019270 | 205.408445 | -0.8063382 | 1.30E-08 | P2ry6 |
| ENSRNOG00000002053 | 204.581428 | -1.0937222 | 8.42E-11 | Fras1 |
| ENSRNOG00000013541 | 204.477459 | -0.8212357 | 2.42E-08 | Sh2d4a |
| ENSRNOG00000029535 | 200.363379 | -1.2039474 | 3.78E-16 | Nrbp2 |
| ENSRNOG00000004078 | 197.391209 | -0.885001 | 1.04E-08 | Eno3 |
| ENSRNOG00000016066 | 196.68327 | -0.9909736 | 6.88E-12 | Bambi |
| ENSRNOG00000051619 | 193.867269 | -1.1526944 | 3.00E-15 | Asb2 |
| ENSRNOG00000016099 | 193.133873 | -2.2919823 | 3.37E-44 | Id4 |
| ENSRNOG00000001825 | 192.58641 | -0.7661113 | 1.12E-06 | AABR07034767.1 |
| ENSRNOG00000027016 | 192.52622 | -0.7996336 | 8.68E-08 | Cobll1 |
| ENSRNOG00000037951 | 192.442125 | -0.8563039 | 2.08E-09 | AABR07039210.1 |
| ENSRNOG00000033740 | 190.503004 | -1.0832767 | 5.76E-13 | Lurap1l |
| ENSRNOG00000021260 | 186.789355 | -1.2330545 | 7.20E-14 | Prnd |
| ENSRNOG00000006789 | 186.63977 | -0.9699356 | 5.17E-11 | Ddit3 |
| ENSRNOG00000012083 | 186.519708 | -1.2227232 | 9.96E-17 | St6galnac2 |
| ENSRNOG00000027489 | 183.858931 | -1.0999159 | 3.65E-13 | Mn1 |
| ENSRNOG00000024082 | 182.829735 | -0.9211305 | 5.87E-09 | Gldn |
| ENSRNOG00000005229 | 182.623092 | -1.008118 | 4.24E-11 | Sec16b |
| ENSRNOG00000024602 | 181.781516 | -1.5722443 | 3.91E-24 | Plekha7 |
| ENSRNOG00000055564 | 177.508566 | -1.4973659 | 3.61E-19 | RGD1564664 |
| ENSRNOG00000004400 | 176.440459 | -1.6817383 | 4.75E-28 | Avpr1a |
| ENSRNOG00000022067 | 175.33772 | -0.9129708 | 8.51E-09 | Tlr5 |
| ENSRNOG00000005573 | 175.324162 | -1.0810127 | 3.09E-13 | Ntn4 |
| ENSRNOG00000015736 | 173.760301 | -0.8543881 | 3.14E-08 | Dhrs3 |
| ENSRNOG00000002227 | 173.056699 | -1.2686729 | 6.98E-14 | Kit |
| ENSRNOG00000025558 | 172.135519 | -1.2277958 | 8.18E-15 | Palm2 |
| ENSRNOG00000023643 | 171.675167 | -1.4789433 | 1.68E-16 | Mmp17 |
| ENSRNOG00000001854 | 171.377706 | -1.7654346 | 5.60E-29 | Tmtc1 |
| ENSRNOG00000025184 | 169.789472 | -2.8509152 | 3.34E-60 | Prss35 |
| ENSRNOG00000009790 | 169.127644 | -1.1411748 | 3.03E-13 | Kcnk3 |
| ENSRNOG00000026941 | 167.78047 | -2.0688076 | 1.51E-29 | Tril |
| ENSRNOG00000022710 | 167.778877 | -0.7504536 | 2.27E-06 | Prrg4 |
| ENSRNOG00000003242 | 167.626384 | -0.7588578 | 3.34E-06 | Gulp1 |
| ENSRNOG00000021812 | 166.02682 | -0.9017746 | 5.77E-07 | Scx |
| ENSRNOG00000011946 | 165.77073 | -1.3794259 | 1.75E-16 | Ptn |
| ENSRNOG00000026059 | 165.706377 | -0.8669084 | 2.88E-08 | Paqr6 |
| ENSRNOG00000020279 | 165.025852 | -0.8941153 | 3.78E-08 | Syt11 |
| ENSRNOG00000033697 | 164.175987 | -1.242258 | 4.41E-16 | Casp4 |
| ENSRNOG00000060542 | 163.783176 | -0.8278089 | 2.00E-07 | Mid2 |
| ENSRNOG00000005930 | 161.387931 | -0.7517509 | 0.00015601 | Nnmt |
| ENSRNOG00000005094 | 160.784659 | -1.1737908 | 2.58E-13 | C1qtnf7 |
| ENSRNOG00000020585 | 158.467601 | -2.2734312 | 8.37E-37 | Tbxa2r |
| ENSRNOG00000047466 | 158.358765 | -0.8194954 | 4.34E-07 | Bdnf |
| ENSRNOG00000002773 | 156.681484 | -3.0250262 | 1.80E-57 | Rgs4 |
| ENSRNOG00000025384 | 156.01887 | -1.1115721 | 2.37E-12 | Slc2a10 |
| ENSRNOG00000059326 | 154.831935 | -0.9887401 | 8.47E-10 | Abca9 |
| ENSRNOG00000013306 | 152.820009 | -1.9213408 | 4.26E-30 | Pcdh20 |
| ENSRNOG00000021256 | 150.782646 | -1.2661646 | 0.0001186 | Adra1d |
| ENSRNOG00000011490 | 150.750115 | -0.8249071 | 4.08E-07 | Angptl8 |
| ENSRNOG00000056184 | 149.297827 | -1.5283078 | 4.69E-18 | Lrp2 |
| ENSRNOG00000004398 | 147.336539 | -2.5164431 | 4.56E-40 | Pkhd1l1 |
| ENSRNOG00000006324 | 144.616258 | -0.8578598 | 1.92E-07 | Trpc6 |
| ENSRNOG00000009204 | 143.652241 | -1.347785 | 1.15E-16 | Il17re |
| ENSRNOG00000004589 | 141.856664 | -1.4606444 | 6.26E-18 | Galnt16 |
| ENSRNOG00000013257 | 141.361212 | -0.876784 | 5.68E-07 | Hecw2 |
| ENSRNOG00000021670 | 139.398193 | -0.9894646 | 8.22E-07 | Frem2 |
| ENSRNOG00000010402 | 138.580747 | -1.8287804 | 8.22E-25 | Hspb2 |
| ENSRNOG00000046366 | 137.506062 | -1.7380086 | 8.32E-25 | Dmd |
| ENSRNOG00000015741 | 136.85448 | -0.8261718 | 3.83E-06 | Slc2a13 |
| ENSRNOG00000007607 | 135.627744 | -0.9668605 | 1.26E-08 | Nr4a1 |
| ENSRNOG00000022871 | 135.535701 | -0.8191565 | 1.09E-05 | LOC691170 |
| ENSRNOG00000019365 | 135.200823 | -1.5843646 | 2.52E-20 | Ablim3 |
| ENSRNOG00000004089 | 134.761754 | -1.0796242 | 2.71E-10 | Enpp2 |
| ENSRNOG00000004488 | 134.728624 | -1.0445782 | 1.08E-09 | Bdkrb1 |
| ENSRNOG00000026573 | 133.655272 | -0.8472164 | 1.22E-06 | Ophn1 |
| ENSRNOG00000018494 | 131.395242 | -1.0012253 | 2.33E-08 | Ppp1r3c |
| ENSRNOG00000013072 | 131.263242 | -1.313838 | 6.65E-12 | Plxna4 |
| ENSRNOG00000005371 | 130.496075 | -1.0797218 | 4.95E-10 | Klhl29 |
| ENSRNOG00000054695 | 126.253895 | -0.8295178 | 3.11E-05 | Calcrl |
| ENSRNOG00000023536 | 125.904092 | -2.1186417 | 1.38E-32 | Adgrd1 |
| ENSRNOG00000048478 | 125.157744 | -1.9796486 | 7.54E-29 | Kcne4 |
| ENSRNOG00000059492 | 124.641725 | -0.8656052 | 0.00015572 | LOC100365363 |
| ENSRNOG00000029394 | 124.615955 | -1.1647331 | 3.72E-10 | Dusp8 |
| ENSRNOG00000013380 | 123.838808 | -0.8970744 | 8.28E-07 | Rhov |
| ENSRNOG00000039336 | 123.590219 | -0.9051076 | 1.27E-06 | Hrct1 |
| ENSRNOG00000013213 | 122.596924 | -0.763211 | 8.53E-05 | Epha4 |
| ENSRNOG00000058609 | 122.585701 | -2.963888 | 3.43E-49 | Palmd |
| ENSRNOG00000023465 | 122.003002 | -1.3604692 | 1.82E-14 | Depp1 |
| ENSRNOG00000024705 | 120.058244 | -1.2902632 | 8.23E-12 | Rarres2 |
| ENSRNOG00000056772 | 118.915383 | -0.9777507 | 2.75E-08 | Col4a6 |
| ENSRNOG00000005673 | 118.80006 | -1.0789336 | 3.53E-09 | Runx1t1 |
| ENSRNOG00000013967 | 118.577677 | -1.0567201 | 7.58E-09 | Blnk |
| ENSRNOG00000016826 | 118.540351 | -0.7538176 | 0.00045582 | Pla2g2d |
| ENSRNOG00000005798 | 117.956705 | -1.8207905 | 1.19E-22 | Cav3 |
| ENSRNOG00000017560 | 117.567682 | -0.8059925 | 5.06E-05 | Mdk |
| ENSRNOG00000011521 | 117.554731 | -1.5928561 | 7.79E-19 | Filip1 |
| ENSRNOG00000052498 | 117.088348 | -1.3106484 | 3.03E-12 | Grb14 |
| ENSRNOG00000003669 | 115.474909 | -1.2686855 | 1.29E-12 | Myocd |
| ENSRNOG00000032618 | 115.363359 | -1.0938296 | 1.03E-09 | Mst1r |
| ENSRNOG00000033434 | 113.807338 | -1.177905 | 3.21E-10 | Casp12 |
| ENSRNOG00000018859 | 113.395556 | -0.7858683 | 9.45E-05 | Pik3ip1 |
| ENSRNOG00000027742 | 111.066345 | -3.5348379 | 5.42E-51 | Adamtsl2 |
| ENSRNOG00000008257 | 110.07457 | -1.542639 | 6.04E-16 | Mfap2 |
| ENSRNOG00000062155 | 110.011002 | -0.7688763 | 0.00082199 | AC134224.2 |
| ENSRNOG00000002548 | 109.808232 | -1.2005628 | 1.31E-09 | Tnn |
| ENSRNOG00000042975 | 109.175211 | -0.9780694 | 2.04E-05 | Tmem45a |
| ENSRNOG00000010350 | 108.867361 | -0.7904949 | 4.50E-05 | Rcan2 |
| ENSRNOG00000021063 | 108.859735 | -0.9189342 | 3.91E-06 | Grin2d |
| ENSRNOG00000010716 | 108.620845 | -1.5686835 | 9.85E-16 | Atoh8 |
| ENSRNOG00000027859 | 107.797687 | -0.9690292 | 5.58E-07 | Tmem26 |
| ENSRNOG00000010685 | 107.776388 | -0.775749 | 6.03E-05 | Tbx18 |
| ENSRNOG00000015353 | 106.964326 | -1.298883 | 1.19E-11 | Prss12 |
| ENSRNOG00000017783 | 105.908212 | -1.167435 | 5.52E-10 | Sfrp1 |
| ENSRNOG00000046271 | 105.584228 | -0.8436203 | 9.25E-05 | Gspt1 |
| ENSRNOG00000028390 | 105.051726 | -0.7605877 | 0.00094945 | Hhipl1 |
| ENSRNOG00000043193 | 104.475331 | -1.0903703 | 1.02E-08 | Smim1 |
| ENSRNOG00000029830 | 103.024024 | -1.0417459 | 9.29E-08 | Adm2 |
| ENSRNOG00000011310 | 102.813106 | -1.8143203 | 4.77E-21 | Pde10a |
| ENSRNOG00000059660 | 101.828194 | -1.044378 | 1.65E-07 | AABR07065531.5 |
| ENSRNOG00000058289 | 101.640375 | -1.0044344 | 1.14E-06 | AABR07027575.1 |
| ENSRNOG00000000156 | 100.54177 | -0.9156054 | 1.78E-05 | LOC100911486 |
| ENSRNOG00000000488 | 14436.5285 | 1.97380025 | 0 | Hmga1 |
| ENSRNOG00000030021 | 14374.7869 | 1.22154477 | 2.57E-81 | Ccl6 |
| ENSRNOG00000005695 | 9393.33428 | 1.00654219 | 3.70E-89 | Mgp |
| ENSRNOG00000008057 | 9326.85419 | 0.78908966 | 4.56E-53 | Krt7 |
| ENSRNOG00000020918 | 8248.74189 | 2.33258239 | 0 | Ccnd1 |
| ENSRNOG00000019587 | 7086.37628 | 1.21939679 | 1.99E-122 | Ptprn |
| ENSRNOG00000046327 | 5867.96642 | 0.98019223 | 4.61E-97 | Rbpj |
| ENSRNOG00000058186 | 5433.79788 | 1.1177597 | 1.37E-108 | Errfi1 |
| ENSRNOG00000002802 | 5409.5907 | 0.82117115 | 3.44E-33 | Cxcl1 |
| ENSRNOG00000002244 | 5306.28035 | 0.76664076 | 7.47E-58 | Pdgfra |
| ENSRNOG00000013720 | 4009.33879 | 0.75046014 | 4.27E-40 | Aebp1 |
| ENSRNOG00000004972 | 3788.7734 | 1.41445598 | 5.94E-138 | Upp1 |
| ENSRNOG00000000047 | 3559.84783 | 0.77156632 | 1.94E-53 | Cd82 |
| ENSRNOG00000028504 | 3375.08543 | 0.80077752 | 1.22E-60 | Socs5 |
| ENSRNOG00000010971 | 3373.35144 | 0.76622602 | 2.71E-36 | Snx18 |
| ENSRNOG00000004276 | 3323.95231 | 1.16829015 | 6.34E-101 | Itga3 |
| ENSRNOG00000055305 | 3191.47542 | 0.95115924 | 2.78E-63 | Parvb |
| ENSRNOG00000026647 | 3182.06366 | 1.19797954 | 1.75E-99 | Cxcl16 |
| ENSRNOG00000012782 | 2464.4724 | 1.03017029 | 2.17E-69 | Tmem2 |
| ENSRNOG00000019924 | 2342.43285 | 0.81442928 | 9.14E-33 | Thop1 |
| ENSRNOG00000021438 | 2336.61041 | 0.91198385 | 1.43E-37 | Tuba1c |
| ENSRNOG00000023896 | 2319.37966 | 1.17393027 | 1.24E-72 | Dusp6 |
| ENSRNOG00000011631 | 2311.80462 | 2.33233729 | 1.02E-264 | Fst |
| ENSRNOG00000017839 | 2211.83719 | 1.67257276 | 2.74E-177 | Ercc1 |
| ENSRNOG00000019996 | 2203.43555 | 0.96357407 | 2.75E-60 | Slc16a1 |
| ENSRNOG00000051163 | 2138.42363 | 1.39917186 | 2.13E-17 | AC114233.2 |
| ENSRNOG00000017671 | 2135.36349 | 0.83913361 | 6.13E-45 | Rasa3 |
| ENSRNOG00000017539 | 2082.02834 | 2.21511816 | 7.12E-224 | Mmp9 |
| ENSRNOG00000002436 | 2069.41032 | 1.97568594 | 7.49E-117 | Mmd |
| ENSRNOG00000006615 | 1973.55453 | 0.91285243 | 1.67E-45 | Mtap |
| ENSRNOG00000001441 | 1892.74608 | 0.87926531 | 7.31E-33 | Tmem120a |
| ENSRNOG00000024688 | 1639.17792 | 2.65758604 | 1.38E-301 | Erfe |
| ENSRNOG00000010516 | 1603.67748 | 0.77062652 | 2.56E-29 | Plau |
| ENSRNOG00000006076 | 1599.29485 | 0.79211907 | 4.54E-36 | Steap2 |
| ENSRNOG00000012852 | 1562.24348 | 0.77137122 | 9.65E-36 | Bin1 |
| ENSRNOG00000028043 | 1560.44023 | 1.71391921 | 7.60E-91 | Cxcl3 |
| ENSRNOG00000000221 | 1538.09309 | 0.76698624 | 3.68E-26 | Abhd5 |
| ENSRNOG00000020703 | 1467.77177 | 0.79761143 | 6.44E-32 | Sipa1l3 |
| ENSRNOG00000019141 | 1422.59067 | 1.68447034 | 2.08E-124 | Ch25h |
| ENSRNOG00000032626 | 1335.31068 | 4.1911393 | 2.59E-136 | Mmp3 |
| ENSRNOG00000020465 | 1317.88959 | 0.79890877 | 8.42E-35 | Ripk3 |
| ENSRNOG00000017704 | 1307.86291 | 0.82449364 | 1.15E-26 | Sema3f |
| ENSRNOG00000015618 | 1303.11688 | 0.91362177 | 3.62E-46 | Wnt5a |
| ENSRNOG00000000567 | 1288.80024 | 1.21034438 | 3.19E-66 | Unc5b |
| ENSRNOG00000020579 | 1272.08169 | 0.95365863 | 6.44E-43 | Col7a1 |
| ENSRNOG00000039668 | 1269.03181 | 1.11642982 | 1.73E-61 | Col8a1 |
| ENSRNOG00000047734 | 1255.58525 | 0.89158061 | 2.18E-35 | Chst2 |
| ENSRNOG00000042460 | 1232.22127 | 3.05607391 | 1.33E-299 | Hmga2 |
| ENSRNOG00000002946 | 1205.50219 | 0.97631342 | 4.74E-44 | Socs3 |
| ENSRNOG00000013851 | 1198.64372 | 1.21933909 | 2.55E-75 | Spry4 |
| ENSRNOG00000005574 | 1175.20787 | 0.98990018 | 7.96E-36 | Adamts8 |
| ENSRNOG00000024728 | 1171.35863 | 1.78671003 | 7.92E-140 | Arhgap22 |
| ENSRNOG00000005347 | 1135.14408 | 0.98071756 | 2.67E-34 | Fjx1 |
| ENSRNOG00000009850 | 1083.65913 | 1.11947481 | 1.64E-52 | St3gal4 |
| ENSRNOG00000006956 | 1045.17954 | 1.164214 | 4.98E-52 | AABR07049085.1 |
| ENSRNOG00000009389 | 1021.9251 | 1.16350103 | 6.29E-53 | Ripk2 |
| ENSRNOG00000003144 | 952.836632 | 1.21603183 | 4.48E-55 | Gprc5c |
| ENSRNOG00000058111 | 946.497969 | 1.64240184 | 1.24E-19 | Itga2 |
| ENSRNOG00000010058 | 941.877359 | 0.78410129 | 3.77E-23 | Spry2 |
| ENSRNOG00000029768 | 890.152043 | 1.31863299 | 4.81E-61 | Ccl12 |
| ENSRNOG00000009369 | 875.445757 | 0.85678004 | 3.96E-21 | Tor4a |
| ENSRNOG00000050224 | 855.790133 | 1.1079478 | 5.83E-42 | Stambpl1 |
| ENSRNOG00000008187 | 851.785855 | 0.78167548 | 1.76E-23 | Ubash3b |
| ENSRNOG00000006320 | 838.825938 | 2.20089728 | 1.80E-151 | Ptges |
| ENSRNOG00000013069 | 823.898297 | 0.89097382 | 2.06E-21 | Sapcd2 |
| ENSRNOG00000020552 | 816.272903 | 1.54188237 | 1.02E-80 | Fosl1 |
| ENSRNOG00000016456 | 786.511729 | 1.83623304 | 2.14E-77 | Il33 |
| ENSRNOG00000027787 | 783.016847 | 0.7513631 | 1.31E-16 | Cdc6 |
| ENSRNOG00000018715 | 774.723897 | 1.30630105 | 1.37E-46 | Clec10a |
| ENSRNOG00000000017 | 742.270285 | 1.22379431 | 1.51E-42 | Steap1 |
| ENSRNOG00000020792 | 703.452615 | 1.70282733 | 3.20E-77 | Etv4 |
| ENSRNOG00000018582 | 691.802667 | 0.83145903 | 9.29E-21 | Exosc6 |
| ENSRNOG00000017850 | 685.927513 | 0.91575048 | 1.49E-26 | Dctpp1 |
| ENSRNOG00000005576 | 674.264438 | 0.86779553 | 3.34E-22 | Rpia |
| ENSRNOG00000015455 | 652.856044 | 0.85685489 | 2.88E-22 | Spr |
| ENSRNOG00000010997 | 648.230757 | 1.91457702 | 2.08E-96 | Ednrb |
| ENSRNOG00000057153 | 637.913572 | 0.79080281 | 2.16E-18 | Pla1a |
| ENSRNOG00000003984 | 624.979727 | 3.17900775 | 1.37E-185 | Apln |
| ENSRNOG00000016013 | 612.682528 | 1.08768419 | 1.42E-34 | Gprc5b |
| ENSRNOG00000054080 | 607.90336 | 1.1791745 | 1.32E-34 | Cgnl1 |
| ENSRNOG00000025527 | 596.479242 | 0.80976084 | 3.74E-18 | Mtcl1 |
| ENSRNOG00000016156 | 590.956995 | 1.3562142 | 2.34E-42 | Nptxr |
| ENSRNOG00000014061 | 589.007003 | 2.10344838 | 3.57E-112 | Dusp5 |
| ENSRNOG00000018874 | 586.226998 | 1.11562423 | 1.05E-37 | Phf19 |
| ENSRNOG00000004956 | 586.048957 | 1.51258198 | 5.98E-57 | Jade2 |
| ENSRNOG00000004854 | 585.447752 | 1.61464921 | 1.05E-56 | Has2 |
| ENSRNOG00000021166 | 582.48775 | 0.93595309 | 1.17E-24 | Ecm1 |
| ENSRNOG00000010797 | 579.614857 | 2.07701418 | 1.41E-23 | Esm1 |
| ENSRNOG00000011205 | 571.93395 | 1.16614412 | 3.54E-38 | Ccl3 |
| ENSRNOG00000000177 | 568.60961 | 0.8277311 | 5.78E-20 | Plpp2 |
| ENSRNOG00000025946 | 568.154955 | 0.77370295 | 4.36E-16 | Igf2bp2 |
| ENSRNOG00000036960 | 548.862534 | 0.93203802 | 4.32E-19 | Abcc9 |
| ENSRNOG00000029614 | 539.689509 | 0.82056705 | 9.65E-19 | Robo1 |
| ENSRNOG00000013304 | 537.493209 | 0.82303816 | 1.69E-18 | Arg1 |
| ENSRNOG00000016246 | 527.577198 | 0.81403326 | 5.28E-17 | Tshz1 |
| ENSRNOG00000013463 | 527.351056 | 1.22381056 | 1.08E-26 | Kcnj8 |
| ENSRNOG00000057404 | 480.353024 | 0.9223659 | 2.60E-17 | Slc47a1 |
| ENSRNOG00000043098 | 478.039016 | 1.91155651 | 4.58E-71 | Mt2A |
| ENSRNOG00000024799 | 470.294636 | 0.82224627 | 6.28E-15 | Cd93 |
| ENSRNOG00000012952 | 455.923801 | 0.75478405 | 2.38E-11 | Lrig1 |
| ENSRNOG00000053430 | 424.763549 | 0.84092802 | 4.84E-15 | Slco4a1 |
| ENSRNOG00000018397 | 414.660329 | 0.98008871 | 4.98E-18 | Dnph1 |
| ENSRNOG00000061519 | 410.028714 | 0.94721614 | 8.08E-16 | Asap2 |
| ENSRNOG00000006030 | 401.73114 | 1.31018097 | 1.79E-34 | Ptprz1 |
| ENSRNOG00000011445 | 386.98007 | 1.01722565 | 1.03E-18 | Nkain1 |
| ENSRNOG00000020105 | 381.740821 | 0.84358049 | 3.37E-14 | Klhl30 |
| ENSRNOG00000051915 | 377.39577 | 0.98075965 | 2.07E-18 | Spred3 |
| ENSRNOG00000009691 | 367.353478 | 2.23495184 | 7.23E-80 | Lrrn2 |
| ENSRNOG00000007561 | 359.151213 | 1.42787239 | 1.04E-32 | Glb1l2 |
| ENSRNOG00000048967 | 340.154382 | 1.95349428 | 3.28E-54 | LOC688459 |
| ENSRNOG00000015366 | 332.567547 | 0.78799096 | 4.02E-10 | Neurl3 |
| ENSRNOG00000026238 | 330.044813 | 0.99043703 | 2.87E-16 | RGD1562618 |
| ENSRNOG00000004575 | 324.936064 | 0.76273501 | 0.01760668 | Il1a |
| ENSRNOG00000053766 | 322.859335 | 0.96014617 | 4.11E-14 | Ramp3 |
| ENSRNOG00000011101 | 310.156613 | 1.26039907 | 3.99E-28 | Twist1 |
| ENSRNOG00000018886 | 301.506989 | 0.77773472 | 1.48E-09 | Aaed1 |
| ENSRNOG00000051056 | 300.452826 | 1.29618512 | 2.23E-26 | AABR07063279.1 |
| ENSRNOG00000048932 | 299.284822 | 0.81420071 | 2.00E-11 | Smagp |
| ENSRNOG00000014197 | 298.104315 | 1.00059554 | 2.06E-15 | Tmem51 |
| ENSRNOG00000013781 | 294.367571 | 0.99869032 | 6.05E-15 | Kcnq5 |
| ENSRNOG00000005708 | 289.618135 | 0.87779434 | 3.82E-12 | Mmp16 |
| ENSRNOG00000005053 | 287.312026 | 1.17613763 | 8.99E-21 | Egln3 |
| ENSRNOG00000023109 | 287.237175 | 1.03203057 | 4.67E-17 | Icoslg |
| ENSRNOG00000019330 | 282.025997 | 0.95594215 | 1.48E-14 | Procr |
| ENSRNOG00000009370 | 281.312376 | 0.84624859 | 7.70E-11 | Tbkbp1 |
| ENSRNOG00000020865 | 277.159114 | 0.80257081 | 8.35E-11 | Ano1 |
| ENSRNOG00000037871 | 245.521458 | 1.14331327 | 2.02E-19 | Sfxn5 |
| ENSRNOG00000008478 | 233.20213 | 5.53087121 | 9.97E-109 | Mmp13 |
| ENSRNOG00000061857 | 232.606238 | 0.90753708 | 5.06E-08 | Mgst2 |
| ENSRNOG00000002792 | 228.565167 | 1.67075094 | 4.64E-31 | Cxcl2 |
| ENSRNOG00000016182 | 227.277272 | 1.76678688 | 4.40E-34 | Tgfa |
| ENSRNOG00000019412 | 227.046712 | 1.0164022 | 6.89E-13 | Rhbg |
| ENSRNOG00000000787 | 222.151609 | 0.84022688 | 9.93E-08 | AABR07044364.1 |
| ENSRNOG00000032832 | 209.712703 | 4.84736689 | 4.31E-101 | Mmp10 |
| ENSRNOG00000007989 | 204.68283 | 1.11445973 | 5.79E-16 | Chst1 |
| ENSRNOG00000000169 | 202.088055 | 0.79095206 | 6.69E-07 | Spata5l1 |
| ENSRNOG00000016326 | 198.458808 | 1.10925629 | 1.13E-13 | Cx3cl1 |
| ENSRNOG00000005227 | 197.339928 | 0.81514382 | 8.98E-08 | Tfap4 |
| ENSRNOG00000048187 | 195.227492 | 0.77919896 | 1.00E-05 | Epop |
| ENSRNOG00000011151 | 195.106668 | 0.81303 | 7.80E-08 | Tenm4 |
| ENSRNOG00000000001 | 187.424234 | 0.87357234 | 2.68E-09 | AABR07013255.1 |
| ENSRNOG00000008525 | 186.522298 | 2.78337389 | 1.16E-60 | Csf3 |
| ENSRNOG00000015160 | 174.643427 | 1.00539123 | 2.67E-08 | Gem |
| ENSRNOG00000060410 | 169.761824 | 0.90481487 | 4.07E-07 | Pcdh1 |
| ENSRNOG00000026653 | 161.24565 | 0.84026107 | 5.29E-07 | Hcar2 |
| ENSRNOG00000017149 | 160.883731 | 1.4271909 | 1.15E-17 | Fam131b |
| ENSRNOG00000007110 | 158.073072 | 0.78882511 | 7.49E-07 | Ankrd6 |
| ENSRNOG00000021276 | 157.340726 | 1.99504639 | 8.63E-35 | Bmp2 |
| ENSRNOG00000019718 | 153.543393 | 1.33299371 | 8.25E-17 | Galnt15 |
| ENSRNOG00000024390 | 151.691988 | 0.81659402 | 5.26E-06 | Osm |
| ENSRNOG00000011406 | 151.103337 | 0.87386539 | 5.99E-07 | Ccl4 |
| ENSRNOG00000009253 | 148.689555 | 1.07913745 | 6.37E-11 | Igsf9b |
| ENSRNOG00000021441 | 148.556897 | 1.18625721 | 5.06E-13 | Reln |
| ENSRNOG00000046848 | 141.222728 | 0.79127403 | 3.26E-06 | PCOLCE2 |
| ENSRNOG00000014357 | 126.55165 | 1.07749723 | 6.85E-10 | Gja4 |
| ENSRNOG00000016753 | 125.299181 | 4.09210392 | 1.80E-65 | Slc14a1 |
| ENSRNOG00000008880 | 120.264579 | 1.1105541 | 5.37E-09 | Nrk |
| ENSRNOG00000026805 | 119.133044 | 1.66654193 | 1.10E-16 | Csf2 |
| ENSRNOG00000013408 | 116.820595 | 2.08651262 | 9.64E-28 | Npas2 |
| ENSRNOG00000022256 | 112.065887 | 2.17306111 | 3.25E-28 | Cxcl10 |
| ENSRNOG00000003384 | 111.646022 | 1.19035196 | 1.28E-08 | Hs3st3b1 |
| ENSRNOG00000017072 | 111.620265 | 0.88439209 | 1.81E-05 | Slc16a14 |
| ENSRNOG00000053384 | 108.272499 | 1.37640246 | 2.96E-12 | Bmp7 |
| ENSRNOG00000000394 | 107.708812 | 0.88227812 | 4.96E-05 | Srgn |
| ENSRNOG00000010598 | 107.449875 | 1.57169243 | 9.64E-17 | Hs3st1 |
| ENSRNOG00000032596 | 106.519494 | 0.76211784 | 7.40E-05 | RT1-T24-1 |
| ENSRNOG00000019584 | 105.305848 | 1.06264014 | 3.30E-08 | Dlk1 |
| ENSRNOG00000011984 | 104.660134 | 2.15696005 | 6.86E-28 | Cxcl14 |
| ENSRNOG00000012098 | 102.881708 | 3.79321227 | 7.17E-55 | Adcyap1r1 |
| ENSRNOG00000015075 | 102.697732 | 1.12447473 | 1.49E-08 | Stc1 |
| ENSRNOG00000017386 | 101.657368 | 1.0610677 | 3.98E-08 | Il11 |
| ENSRNOG00000043486 | 100.463326 | 0.96193219 | 9.68E-07 | Tnfrsf26 |


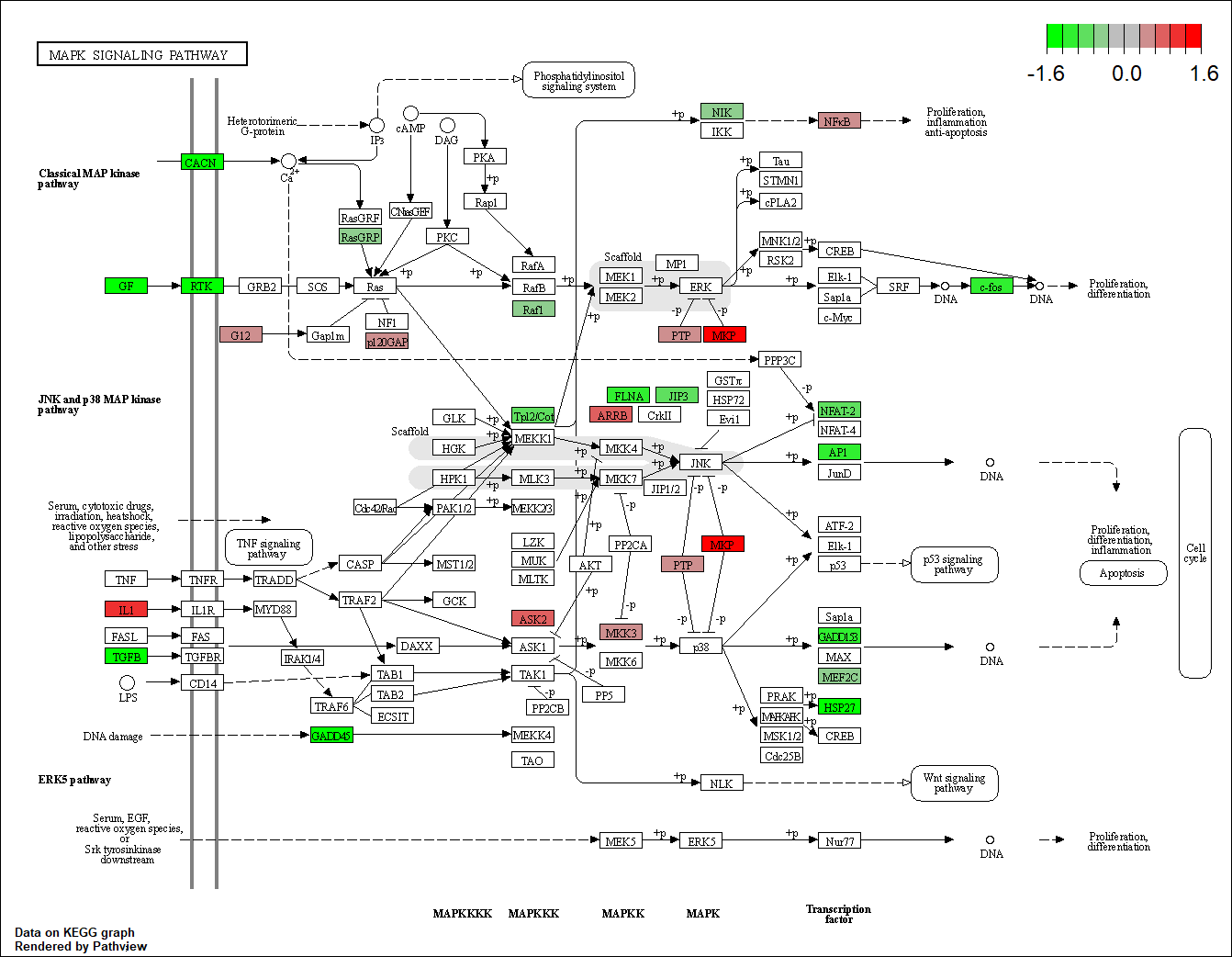
**Figure S1. The genes in MAPK signaling pathway.**

**Figure S2. The genes in PI3K-AKT signaling pathway.**


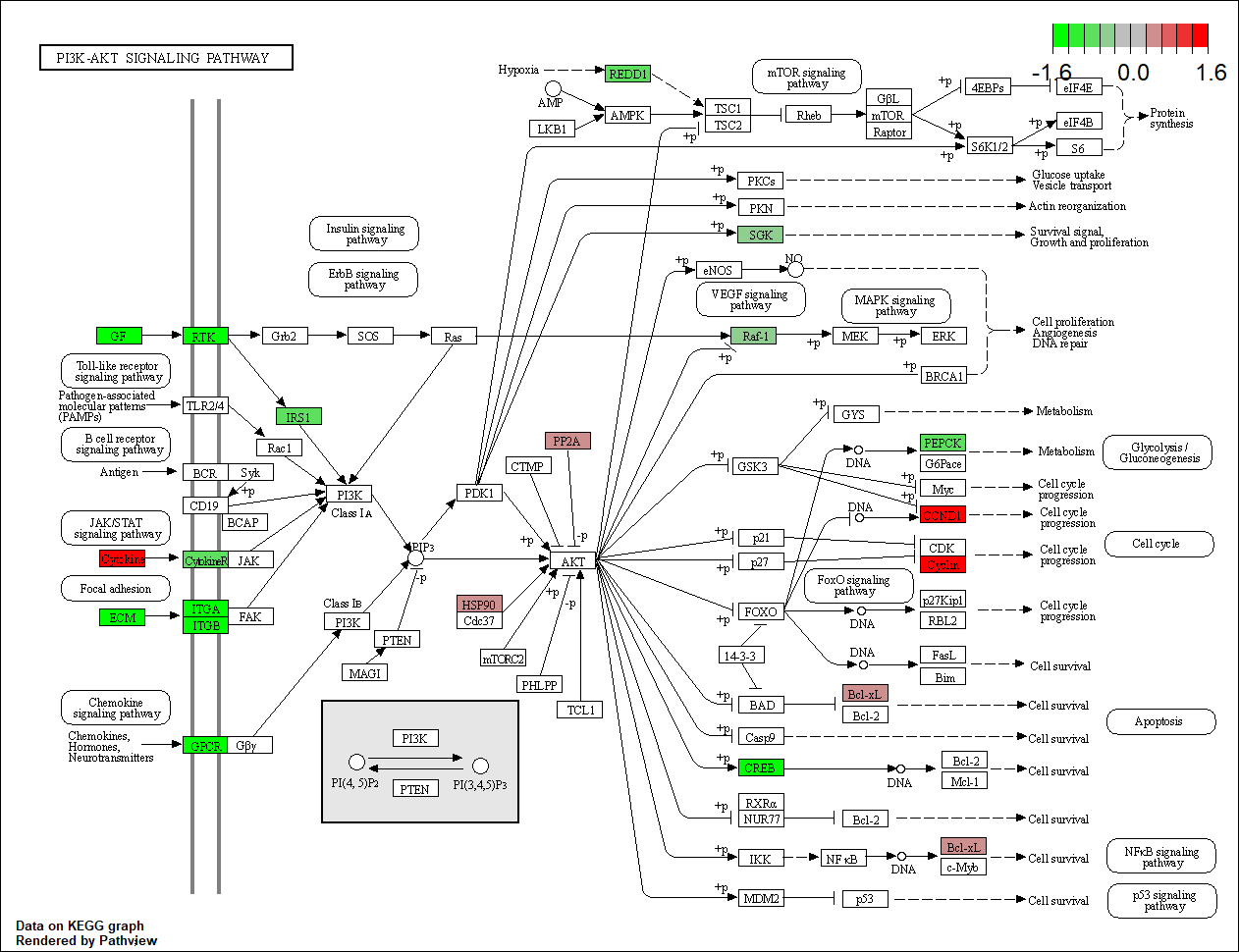


**Figure S3. The genes in WNT signaling pathway.**


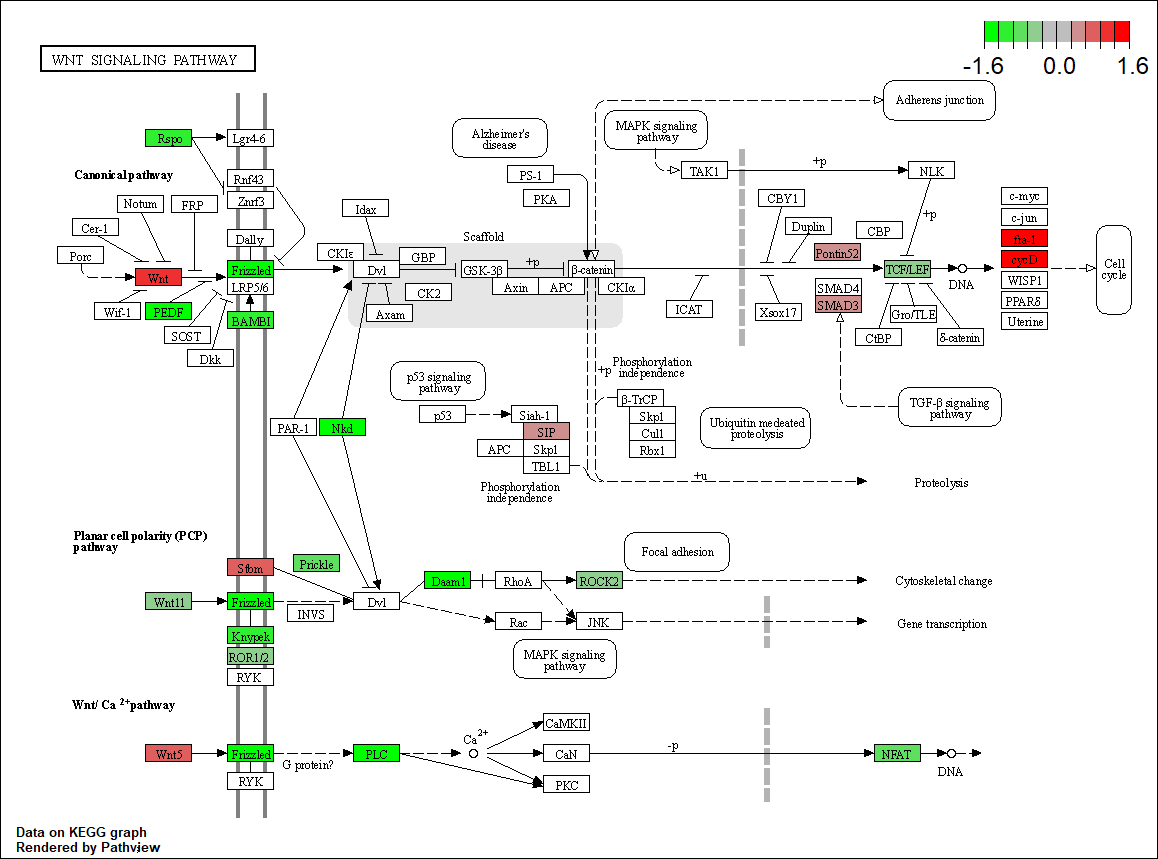


**Figure S4. The genes in Focal adhesion signaling pathway.**


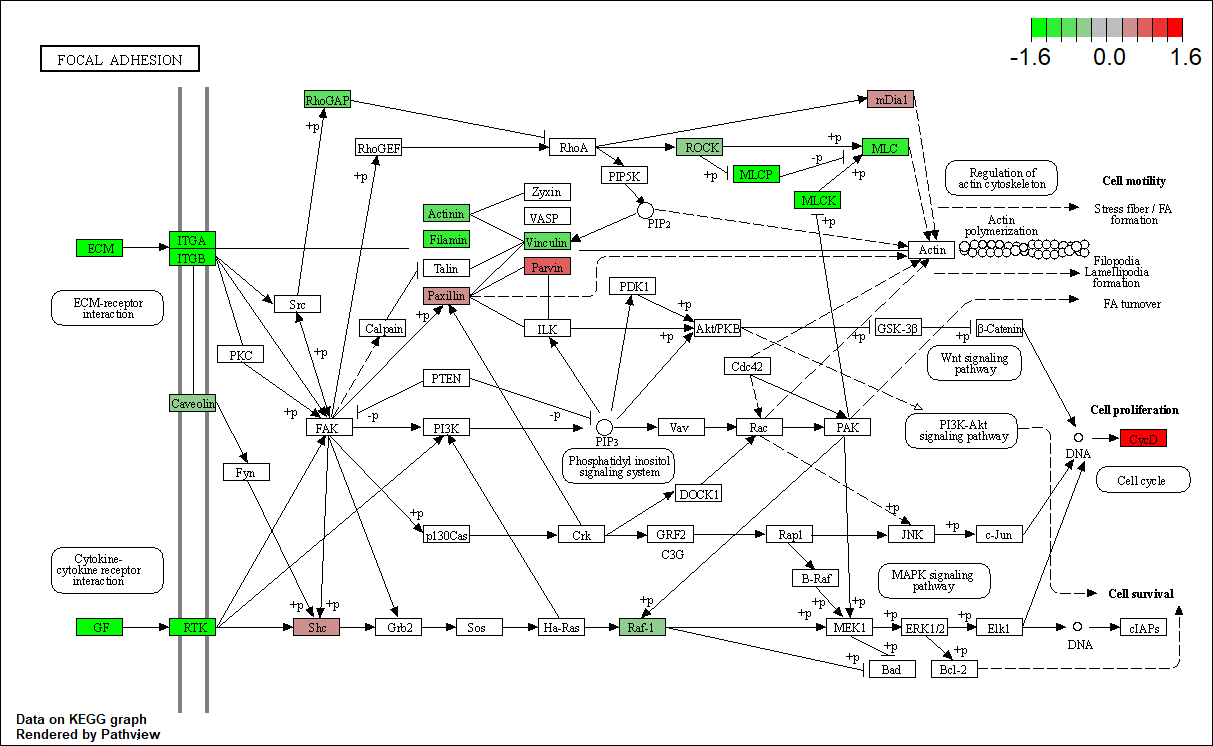

Supplement: Supplementary file 1 — Fig. S1. Genes in MAPK signaling pathway. Fig. S2. Genes in PI3K‐AKT signaling pathway. Fig. S3. Genes in WNT signaling pathway. Fig. S4. Genes in Focal adhesion signaling pathway. Table S1. Oligonucleotide primers for qRT‐PCR in this study. Table S2. Significantly up or downregulated DEGs. [file FEB4-13-1895-s001.docx]
